# Morphotype Transition and Sexual Reproduction Are Genetically Associated in a Ubiquitous Environmental Pathogen

**DOI:** 10.1371/journal.ppat.1004185

**Published:** 2014-06-05

**Authors:** Linqi Wang, Xiuyun Tian, Rachana Gyawali, Srijana Upadhyay, Dylan Foyle, Gang Wang, James J. Cai, Xiaorong Lin

**Affiliations:** 1 Department of Biology, Texas A&M University, College Station, Texas, United States of America; 2 Department of Veterinary Integrative Biosciences, Texas A&M University, College Station, Texas, United States of America; University of California, San Francisco, United States of America

## Abstract

Sexual reproduction in an environmental pathogen helps maximize its lineage fitness to changing environment and the host. For the fungal pathogen *Cryptococcus neoformans*, sexual reproduction is proposed to have yielded hyper virulent and drug resistant variants. The life cycle of this pathogen commences with mating, followed by the yeast-hypha transition and hyphal growth, and it concludes with fruiting body differentiation and sporulation. How these sequential differentiation events are orchestrated to ensure developmental continuality is enigmatic. Here we revealed the genetic network of the yeast-to-hypha transition in *Cryptococcus* by analyzing transcriptomes of populations with a homogeneous morphotype generated by an engineered strain. Among this network, we found that a Pumilio-family protein Pum1 and the matricellular signal Cfl1 represent two major parallel circuits directing the yeast-hypha transition. Interestingly, only Pum1 coordinates the sequential morphogenesis events during **a**-α bisexual and α unisexual reproduction. Pum1 initiates the yeast-to-hypha transition, partially through a novel filament-specific secretory protein Fas1; Pum1 is also required to sustain hyphal growth after the morphological switch. Furthermore, Pum1 directs subsequent differentiation of aerial hyphae into fruiting bodies in both laboratory and clinical isolates. Pum1 exerts its control on sexual reproduction partly through regulating the temporal expression of Dmc1, the meiosis-specific recombinase. Therefore, Pum1 serves a pivotal role in bridging post-mating morphological differentiation events with sexual reproduction in *Cryptococcus*. Our findings in *Cryptococcus* illustrate how an environmental pathogen can ensure the completion of its life cycle to safeguard its long-term lineage success.

## Author Summary

Sex, despite its cost, is an important means to maximize species fitness in coping with unpredictable environmental challenges. In the human fungal pathogen *Cryptococcus neoformans*, sexual reproduction has yielded hyper virulent and drug resistant variants, and produces airborne infectious spores. Developmentally, sexual spores are generated from fruiting bodies that are differentiated from aerial hyphae. *Cryptococcus* cells typically grow as yeast cells with a subpopulation that respond to mating stimulation and switch to hyphal growth after mating. However, mechanisms that connect sexual reproduction and multiple differentiation events to ensure the developmental continuality are unknown. Here we revealed a network of yeast-to-hypha transition in *Cryptococcus*. From this network we identified a Pumilio-family RNA binding protein Pum1 that acts in concert with the matricellular signal Cfl1 in regulating the yeast-to-hyphal transition following mating. Interestingly, Pum1 is also important in sustaining hyphal growth and in directing the progression from aerial hyphal morphogenesis to the formation of fruiting bodies. Intriguingly, mutations of Pum1 affect the spatiotemporal expression pattern of the filament- and meiosis-specific proteins Fas1 and Dmc1. Our study opens a new avenue to investigate how a microbe controls development continuity while maintaining population heterogeneity.

## Introduction

Selective pressures from the environment shape microbial evolution. To cope with the challenges presented by both predictable and erratic environmental fluctuations, microbes employ various adaptation and bet-hedging strategies, like coordinated community behaviors and morphotype transition [1], [2], [3], [4], [5], [6], [7]. The transition between different morphotypes confers genetically identical cells the distinct ability in responding to different environmental stimuli. This maximizes the community fitness and enhances species survival under disparate conditions [3], [4], [6], [8], [9]. Similarly, morphotype transition is widely adopted by evolutionally divergent pathogens to assist their survival both inside and outside of the host [3], [4], [6], [7], [9]. The causal agent of the most common fungal disease of the central nervous system, *Cryptococcus neoformans*, can undergo the transition between yeast and hypha states [6], [10]. This morphotype transition is linked with its virulence potential [7].

Hyphal growth (filamentation) in this ubiquitous pathogen generally occurs as a cellular response to environmental stimuli that induce sexual reproduction [11], [12], [13]. Sexual reproduction in *Cryptococcus* has been known for decades to take place between cells of opposite mating types, **a** and α (bisexual mating). Such bisexual mating generates an equal number of **a** and α meiotic progeny [14], [15]. However, the *Cryptococcus* population worldwide is sharply skewed towards the α mating type (>99%) and the chance of locating a compatible mating partner nearby is slim. Yet, many natural and clinical isolates maintain the ability to mate [14]. The discovery of the unisexual life cycle in *C. neoformans* that involves cells of only a single mating type, most often the α mating type [16], [17], [18], offers a plausible explanation for the observed dominance by α isolates [12], [16], [17], [19]. Besides the fact that spores produced by sexual reproduction are infectious propagules [20], [21], unisexual mating may have played a variety of roles in cryptococcal infections [22], [23], [24]. For instance, α unisexual mating is proposed to have yielded hyper-virulent *Cryptococcus* isolates [22], and may have assisted this pathogen in adapting to host environment [25]. Unisex in *C. neoformans* can also lead to aneuploidy [24], which could offer fitness benefit under certain stress conditions [26].

**Figure 1:**
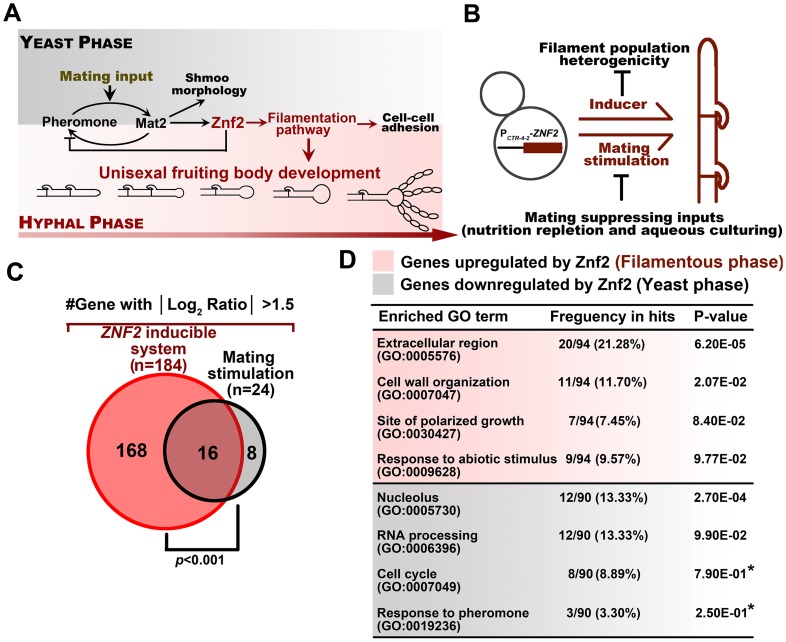
Identification of the genetic network in hyphal and yeast populations. (A) A diagram depicting the pheromone and filamentation signaling pathways and their downstream cellular processes in *Cryptococcus*. (B) The P*_CTR4-_2-ZNF2* strain and the wildtype strain H99 were grown under the condition that inhibits mating activation in order to perform comparative transcriptome analyses of homogeneous yeast and hyphal populations without the complication pheromone stimulation. (C) Histogram and the Venn diagram depicting the difference in two transcriptome analyses. The current one (red) used the homogeneous morphotypes and yielded a higher number of genes with significantly differential expression between the yeast and filamentous growth. The former one (black) used wildtype population in response to mating stimulation and the population showed pronounced heterogeneity in cell morphotype, which likely obstructed the previous effort in identifying many of the potential Znf2 downstream targets. The significant overlap of the lists from this study and the previous one verified our current approach and also indicated some factors that are likely to be specific to the growth conditions (hypergeometric *p*-value of significant overlap <0.001). (D) The list of enriched GO terms with differentially expressed genes in different morphotypes. The DAVID gene ontology program was used for the analysis and gene-enrichment in the annotated terms was evaluated based on the EASE Score. The EASE Score is a modified Fisher Exact P-Value and the default threshold is 0.1 [29]. Asterisks indicate GO terms with values above the default threshold. These GO terms are included because of their potential biological significance based on previous studies in *C. neoformans* or other fungi [11], [12], [30], [47]. The extremely mating-suppressive conditions used in this study may result in the under-representation of these classes and their lower statistical significance.

Sexual reproduction, including both unisexual and bisexual reproduction, occurs only in a subpopulation of a *Cryptococcus* mating community. It involves sequential morphological differentiation events in a stochastic manner [23]. The life cycle of this pathogen commences with early mating events controlled by the pheromone signaling pathway and is followed by the transition from the yeast to the hyphal form. Hyphae generated from this morphological transition are mostly concentrated at the periphery of the mating colony and they can invade solid substrates, extend on the surface, or expand into air. Some of the aerial hyphae further develop into fruiting bodies on their apexes, which form cup-shaped basidia that eventually give rise to four chains of spores (Figure 1A). The spores disperse and germinate into yeasts and a new life cycle begins.

The architecture of the upstream signaling pathways engaged in unisexual reproduction resembles that of bisexual reproduction [11], [16]. In this regard, the pheromone signaling (MAPK pathway) plays a prominent role in integrating external inputs into the initiation of the mating development [11], [23], [27] (Figure 1A). The ultimate decision maker for the morphological transition from yeasts to hyphae is the C2H2 zinc finger regulator Znf2. Upregulation of Znf2 initiates the formation of dikaryotic (bisexual) or monokaryotic (unisexual) hyphae and Znf2 is also required to sustain hyphal growth [18]. Overexpression of Znf2 can drive the yeast-to-hypha transition independent of environment cues, which generates a homogenous hyphal population [7]. Thus, Znf2, as a master regulator of filamentation, links the upstream signaling activation, including the ones from the pheromone pathway, to the yeast-hypha cellular response.

Like many other human fungal pathogens, the yeast-to-hypha transition is linked with virulence potential in *Cryptococcus*, and Znf2 plays a crucial role in this connection [7], [28]. Znf2 orchestrates these two behaviors (morphogenesis and virulence) partially through its downstream target Cfl1 [7]. Cfl1 is a cell-wall associated adhesion protein and it also functions as a signaling molecule upon its release into the extracellular matrix [2]. This matrix protein plays a similar but less prominent role than Znf2 in regulating filamentation in *C. neoformans* [7]. These observations indicate the existence of additional players in coordinating hyphal development.

In this study, we elucidated a genetic network controlling morphotype transition in *C. neoformans*. We discovered that Pum1, a RNA-binding protein, acts in concert with Cfl1 to direct Znf2-dependent filamentation. Pum1 plays a pleiotropic role in cryptococcal development: it regulates the initiation and the extension of hyphal growth; it directs the progression from aerial hyphal morphogenesis to the formation of fruiting body; and intriguingly, it also controls meiosis and sporulation during bisexual and unisexual mating. Pum1 regulates filamentation and meiosis partly through its control of the spatiotemporal expression pattern of filament-specific and meiosis-specific proteins Fas1 and Dmc1. Not surprisingly, Pum1 is critical for filamentation and sporulation in both laboratory and clinical isolates. Hence, this investigation offers a new prospective in our understanding of forces that shape cell fate and sexual reproduction in environmental pathogens.

## Results

### Identification of potential components in the filamentation pathway using an engineered strain that produces a homogeneous hyphal population

We previously demonstrated that Znf2 is the decision maker of filamentation in *Cryptococcus* and it is sufficient and necessary to direct hyphal formation [7], [11], [28]. Firstly, Znf2 is required for initiating filamentation and the deletion of *ZNF2* locks cells in the yeast form [11]. Secondly, the expression level of *ZNF2* is positively correlated with the amplitude of filamentation [7], [28]. Thirdly, overexpression of *ZNF2* stimulates robust filamentation irrespective of environmental stimulation or pheromone signaling [7]. Lastly, attempts of UV mutagenesis in the *znf2*Δ mutant background failed to recover any suppressor mutations that could restore hyphal growth (data not shown). By comparison, from the 60,000 mutants that we screened (19 Mb genome, ∼7,000 genes), mutants that could form other cell types (e.g. pseudohypha or shmoo) were isolated. Given the central role of Znf2 in filamentation, we hypothesize that Znf2 coordinates the expression of multiple factors that are critical for hyphal development. Thus, characterization of Znf2 regulon is expected to provide a global insight into the molecular bases for hyphal development.

Filamentation in *C. neoformans* usually occurs in response to mating cues (Figure 1B) and the morphological state in a mating community is highly heterogeneous (Figure S1). Even in a mature mating colony, the yeast sub-population dominates over the hyphal sub-population (Figure S1). Such heterogeneity in morphotype presents a challenge to identify filamentation-specific molecules. To circumvent this complication, we took advantage of a strain with the *ZNF2* gene under the control of the promoter of the copper transporter gene *CTR4* (P*_CTR4-2_-ZNF2*) [7]. The proportion of filaments generated by this P*_CTR4-2_-ZNF2* strain is positively correlated to the degree of copper-limitation, which induces its expression [7]. To eliminate potential noise caused by other cell types (e.g. shmoo cells) generated in response to mating-inducing cues, we cultured this engineered strain under conditions that are known to be extremely mating-suppressive [7]. Under such conditions, the P*_CTR4-2_-ZNF2* strain generated a nearly homogenous filamentous population (Figures 1B and S1).

We then compared the transcription profiles of the two populations with homogeneous morphotypes cultured under the above mentioned conditions: the wild type (yeasts) and the P*_CTR4-2_-ZNF2* strain (hyphae) (Figure S1). The genes that were differentially expressed between the two morphotypes (populations) were selected. A much larger number of differentially expressed genes were identified with a wider range of expression amplitudes by this current approach compared to the previous data generated from a comparison between the wildtype and the *znf2*Δ mutant in response to mating stimulation (Figure S1) [11]. This current approach of using the two morphologically homogenous populations allowed us to identify 184 genes that exhibited more than 3 fold differences in the expression level (Figure 1C and Table S1). In comparison, only 24 genes above that threshold were identified by our previous approach [11]. Although different culture conditions were used, we believe that the lower sensitivity of the previous approach was largely attributable to the morphological heterogeneity of the mating-colony (Figure S1). Thus, this current approach provides us with a more comprehensive regulon of Znf2.

Functional prediction of this expanded regulon indicates that genes related to secretion and cell wall organization are the two functional groups overrepresented in the filamentous state (Figure 1D). As expected, some genes that are highly induced by Znf2 were also identified and confirmed in our previous study [7]. A gene ontology analysis of the new data by the DAVID program [29] revealed additional biological processes associated with Znf2-stimulated filamentous growth, including polarized growth and stress responses (Figure 1D). Both are also implicated in filamentous growth in other dimorphic fungi [30], [31], [32]. “Response to pheromone” was also identified as a distinctive class, although this class was not found to be statistically significant (Figure 1D). However, given that the condition used for the transcriptome experiment was extremely suppressive to the pheromone sensing pathway or mating [7], we speculate that this identification is biologically significant even though the *P*-value for this category might be underwhelming. This finding likely reflects the unique feature of *Cryptococcus* among human fungal pathogens in that filamentation in this organism is typically a cellular response to mating signals. The indication that Znf2, a filamentation master regulator, may actually repress pheromone signaling genes is seemingly contradictory to the above notion. However, it is consistent with an inhibitory effect of Znf2 on the expression of early mating genes and early mating events noted in two earlier studies [11], [12]. Collectively, these observations suggest negative feedback from hyphal growth on the mating stimulation that initiates the hyphal formation. Such prohibition of cell fusion and pheromone-response during hyphal differentiation could be important to confine the cell lineage for the proper development progression.

### Pum1 is an important regulator in the filamentation pathway

**Figure 2:**
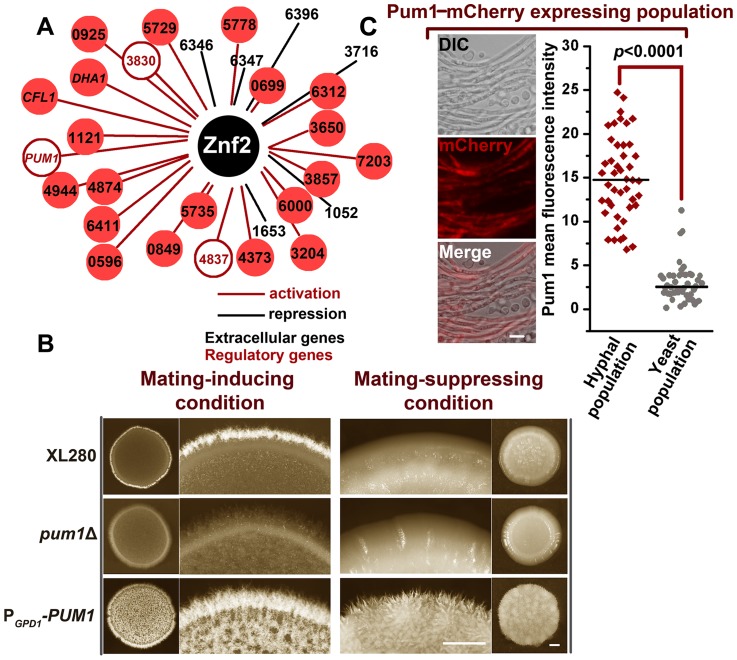
The Znf2 downstream target Pum1 directs filamentation. (A) The 29 Znf2 downstream targets were placed under the control of the constitutive P*_GDP1_* promoter to construct a *Cryptococcus* overexpression library. The screen for their effects on filamentation was conducted under filamentation-suppressing or filamentation-inducing conditions with a 5-day incubation period. CNAG_0‘number’ indicates the gene locus name used in Broad Institute *C. neoformans* H99 sequence annotation, and these locus names were used because they are previously uncharacterized genes. The gene locus number of *PUM1* is CNAG_04003. (B) Pum1 positively controls filamentation. The overnight cultures of the wildtype strain XL280, the *pum1*Δ mutant, and the *PUM1* overexpression strain of the same cell density were dropped onto the V8 agar (mating-inducing) or the YPD agar (mating-suppressing) and photographed after 5 days of growth. Scale bar: 1 mm. (C) The Pum1-mCherry shows a biased expression in the hyphal subpopulation. The images of the fluorescence labeled wildtype strain were taken at 72 hrs post mating stimulation. The localization patterns of Pum1 in the figure are representative for each given cell types (at least 40 cells for each cell type were examined). See *Materials and Methods* for the detailed description of the experimental condition for the sub-cellular localization of Pum1-mCherry. Scale bar: 10* µ*m.

To assess the biological roles of Znf2 targets in filamentation, we chose to overexpress part of the Znf2 regulon (29 genes) that encode potential regulators or extracellular products (Figure 2A). To identify genes that are specifically involved in filamentation but not important in activating pheromone signaling, we screened this overexpression library for filamentation on YPD medium, a culture condition that suppresses mating behaviors [7]. We found that overexpression of CNB04870 (serotype D JEC21/XL280 background) resulted in a robust filamentation phenotype under the mating-suppressing condition (Figure 2B). Conversely, the deletion of this gene dramatically reduced filamentation in the hyper-filamentous XL280 background under filamentation-inducing condition (Figure 2B). Thus both the gain of function (overexpression) and the loss of function (gene knockout) demonstrated the significance of this gene in initiating filamentation. Motif searches using the Motif Scan and the InterProScan programs revealed a CLN-rich region at its N-terminus and a Pumilio-family RNA binding domain at its C-terminus (Figure S2A). The Pumilio domain is conserved throughout the eukaryotic domain and its family members are known for their regulatory roles in a wide range of processes, including mitochondrial biogenesis, cell division, development, and differentiation [33], [34]. We therefore named this gene *PUM1* (Pumilio protein 1) (Figure S2A). Consistent with the idea that *ZNF2* regulates *PUM1*, the expression of *PUM1* was considerably increased by the overexpression of *ZNF2* (Figure S2B). To examine the cell type that expresses Pum1 during the development of a mating community, we fused the fluorescence protein mCherry with Pum1 that is driven by the *PUM1*'s native promoter. In agreement with the notion that Pum1 belongs to the Znf2-controled filamentation pathway, Pum1 is expressed in the hyphal population at a significantly higher level than that in the yeast population (Figure 2C).

**Figure 3:**
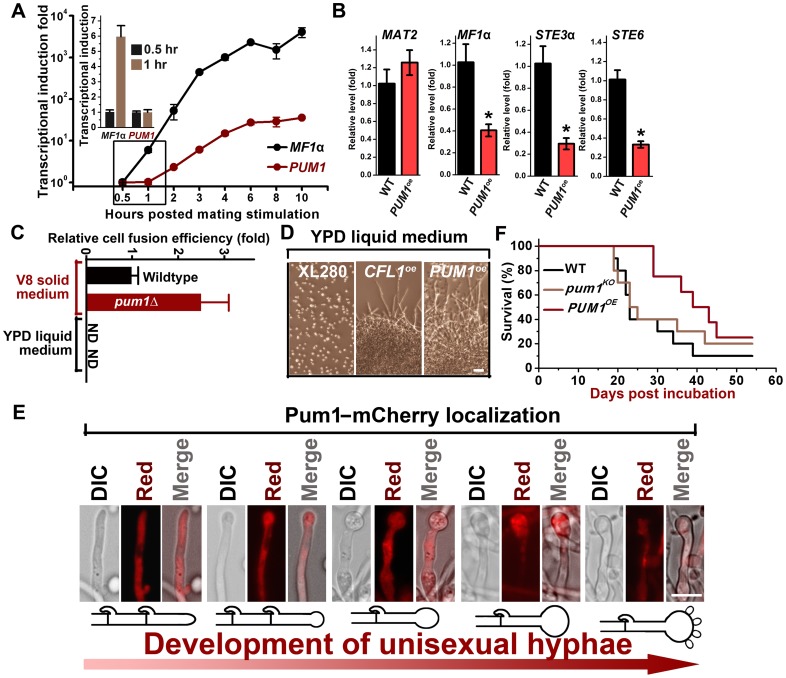
Pum1 controls filamentation but not early mating processes. (A) Transcriptional dynamics of *MF1*α and *PUM1* during mating. The RNA from the **a**+α mixture co-cultured on V8 agar at 22°C in the dark at indicated time points was subjected to the transcriptional analysis by real time PCR. There was a delay in the transcriptional induction of *PUM1* compared to that for the pheromone gene *MF1*α. (B) Overexpression of *PUM1*did not increase the transcript level of the genes in the pheromone pathway. The expression level of the corresponding genes in the wildtype strain XL280 grown under the same condition was arbitrarily set as 1 for comparison. (C) Pum1 is modestly inhibitory for cell fusion. The α and **a** partners from wildtype strains or the *pum1*Δ mutant marked with different auxotrophic markers were co-cultured on V8 agar or YPD liquid medium for 22 hours at 22°C in the dark. The cocultures were then collected, washed, and plated onto YNB minimal medium to select for fusion products. The cell fusion efficiency of the wildtype coculture was arbitrarily set as 1 for comparison. (D) Overexpression of *PUM1* and *CFL1* are sufficient to trigger filamentation even under mating-suppressive conditions. The wildtype strain, the P*_CTR4-2_-PUM1* strain, and the P*_CTR4-2_-CFL1* strain were grown in the YPD liquid medium. Filamentation and cell aggregation (cell-cell adhesion) were observed in the *CFL1^oe^* and *PUM1^oe^* strains but not in the wildtype strain. Scale bar: 20* µ*m. (E) The P*_GPD1_-PUM1* strain was significantly less virulent in a murine intranasal inhalation infection model compared with the wildtype strain XL280 and the *pum1*Δ mutant (*P*<0.01). (F) Pum1-mCherry is expressed at a relatively constant level throughout hyphal growth and basidial development (>40 hyphae for each stage were examined). Scale bar: 10* µ*m.

It is known that Znf2 and its previously characterized downstream factor Cfl1 do not control filamentation through feedback activation of the upstream pheromone signaling circuit or early mating events [2], [11]. Rather, Znf2 and Cfl1 are dedicated morphogenesis/filamentation regulators [2], [11]. Pum1 is also a filamentation regulator, as overexpression of *PUM1* initiated hyphal formation even under the condition that was extremely inhibitory to mating activation (Figure 2B and Figure 3D). The observation that the transcriptional induction of *PUM1* was delayed compared to early mating genes (e.g. the pheromone gene *MF1*α) during mating development (Figure 3A), as we previously observed for *ZNF2* and *CFL1* [2], [7], led us to speculate that Pum1 is also a filamentation specific regulator and it is not critical for early mating events. Indeed, the overexpression of *PUM1* did not increase the expression of *MAT2*, a key transcription factor that controls pheromone sensing and response [11](Figure 3B). On the contrary, we observed a reduction in expression of the early mating genes that are involved in the synthesis (*MF1*α), secretion (*STE6*), and response (*STE3*) of the pheromone signal in the *PUM1* overexpression strain (Figure 3B). This finding echoes the inhibitory effect of Znf2 and Cfl1 on the early mating genes [2], [11] and suggests that Pum1 is likely involved in suppressing the pheromone circuit in an inhibitory feedback manner. Consistently, the deletion of *PUM1* enhanced cell fusion efficiency, an early cellular response to mating induction (Figure 3C).

To study Pum1's spatiotemporal regulation of filamentation, we examined the localization of Pum1-mCherry driven by its native promoter throughout hyphal development (Figure 3E). Pum1 exhibited a relatively constant expression level during the progression of hyphal development, and the majority of Pum1 proteins were localized in the cytoplasm. Such subcellular localization of Pum1 is consistent with its predicted function as a mRNA-binding protein [33]. Intriguingly, Pum1 accumulated at the aerial hyphal apexes where basidia form upon the initiation of fruiting body development (Figure 3E). An intense patch of Pum1-mCherry was observed in the basidial heads and the signal gradually became dimmer after spore formation. The spatiotemporal expression pattern of Pum1 implicates its involvement in the regulation of fruiting body formation in addition to its role in filamentation.

Morphotype has long been associated with virulence in *Cryptococcus*, with the yeast form being pathogenic and the filamentous form attenuated in virulence [11], [28]. Our previous study demonstrated that Znf2 links cryptococcal morphotype and virulence potential. The deletion of *ZNF2* locks cells in the yeast phase and enhances virulence while the overexpression of *ZNF2* leads to filamentation *in vivo* and abolishes cryptococcal virulence in the clinical isolate H99 background and significantly attenuates the virulence in the XL280 background in a mouse model of cryptococcosis [7], [28]. Given that Pum1 regulates filamentation, we decided to test the impact of mutations of Pum1 on cryptococcal virulence. Here we examined the wildtype XL280, the *pum1*Δ mutant, and the P*_GPD1_*-*PUM1* strain in the inhalation infection model of murine cryptococcosis. The P*_GPD1_-PUM1* strain harbors the *PUM1* gene driven by the promoter of the house keeping gene *GPD1*. This strain was considerably attenuated in pathogenicity (Figure 3F). The comparable level of virulence between the wildtype and the *pum1*Δ mutant was likely due to a low level of expression of Pum1 in the wildtype during infection and it is known that mammalian physiological conditions are extremely inhibitory to the filamentation program [7]. Consistently, the Pum1-mCherry was below detectable level under the conditions that mimic host environments.

### Pum1 and Cfl1 represent two branches downstream of Znf2 in controlling aerial structure development

**Figure 4:**
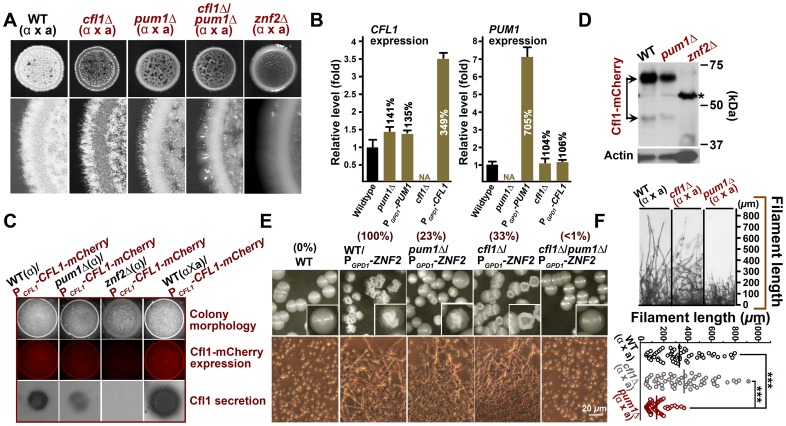
Cfl1 and Pum1 represent two major circuits downstream of Znf2 in directing aerial hyphae formation. (A) Transcriptional analysis by qPCR indicates that alterations of Cfl1 or Pum1 do not affect the transcript level of each other. The expression level of genes in the wildtype strain XL280 was arbitrarily set as 1 for comparison. (B) The production or the release of Cfl1 proteins was not drastically affected in the *pum1*Δ mutant. The expression of Cfl1 proteins was abolished in the *znf2*Δ mutant and enhanced when the compatible mating partner was present. The mCherry-labeled Cfl1 was used to measure the Cfl1 protein level by fluorescence microscopy and released Cfl1 proteins was detected by colony immunoblot. (C) Pum1 is dispensable for the expression or the processing of Cfl1. By comparison, the Cfl1 protein expression was below the detectable level in the *znf2*Δ mutant. This served as a negative control. The asterisk indicates the non-specific band, which is also shown in control strain XL280 without mCherry. (D) Double deletion of *CFL1* and *PUM1* resulted in a much more severe defect in aerial filamentation during bisexual mating compared to either of the single deletion. (E) Pum1 and Cfl1 represent two major downstream regulatory branches of Znf2 in initiating filamentation. (F) Pum1 is also involved in hyphal extension. The deletion of *PUM1* resulted in shorter filaments during bisexual mating. Such a phenotype was not observed in the *cfl1*Δ mutant. Scale bar: 20* µ*m.

During the development of a mating colony, a portion of cryptococcal cells switch from the yeast to the hyphal form and subsequently generate two types of hyphae: invasive hyphae and aerial hyphae [7]. Invasive hyphae penetrate and grow beneath the solid substrate in search of nutrient while aerial hyphae expand into the air for reproduction and spore dispersal. Znf2 is required for the formation of both types of hyphae [7]. Surprisingly, the deletion of *PUM1* considerably reduced the formation of aerial hyphae but exerted a much more modest effect on invasive filamentation under different conditions (Figures 4A, S3A and S3B). Interestingly, the previously characterized extracellular matrix signal protein Cfl1 [2] is also more critical for aerial hyphal development than for invasive hyphal growth (Figure S3A). The similar effect of Pum1 and Cfl1 on the three-dimensional architecture of a mating colony prompted us to further investigate their genetic interactions.

To interrogate the relationship between Pum1 and Cfl1, we first examined that the reciprocal effect of the disruption or the overexpression of one gene on the transcript level of the other. We did not observe any significant changes in expression of one gene when the other gene was altered (deletion or overexpression) (Figure 4B). Thus it appears that Cfl1 and Pum1 do not modulate each other at the transcript level in a major way. We next examined the effect of Pum1 on the abundance of Cfl1 protein within a community. We introduced Cfl1-mCherry under the control of *CFL1*'s native promoter into the wildtype strain, the *pum1*Δ mutant, and the *znf2*Δ mutant. As expected, the Cfl1 protein expression was almost abolished in the *znf2*Δ mutant (Figure 4C). By comparison, the Cfl1 protein expression level in the *pum1*Δ mutant was only slightly affected, suggesting that Pum1 has no major impact on the protein level of Cfl1. Western blot analyses further supported this conclusion (Figure 4D). Thus, unlike Znf2, Pum1 is not required for the expression of the Cfl1 protein.

We previously established that Cfl1 is secreted extracellularly and that some of the Cfl1 proteins are cleaved and released into the surrounding milieu [2]. The released Cfl1 proteins can induce the expression of endogenous Cfl1 in nearby cells and stimulate their filamentation in a paracrine manner [2]. Thus an impairment of Cfl1's secretion or its release could lead to deficient filamentation, a phenotype observed in the *pum1*Δ mutant. To assess the impact of the deletion of *PUM1* on the secretion and releasing of Cfl1 from the cell wall, we employed the colony immunoblot assay, which detects released proteins from an intact colony [2]. As shown in Figure 4C (bottom panel), released Cfl1 was not detected in the *znf2*Δ mutant while the deletion of *PUM1* only modestly reduced the level of released Cfl1 proteins. Furthermore, some of the Cfl1 proteins were correctly cleaved into a smaller product in the *pum1*Δ mutant as in the wildtype strain (Figure 4D). Taken together, these observations suggest that Pum1 is not critical for the expression, secretion, or the processing of Cfl1. Because the *pum1*Δ *cfl1*Δ double mutant displayed a much more severe defect in aerial hypha development than either of the single mutant alone (Figure 4A), Pum1 and Cfl1 represent two parallel branches modulating Znf2-mediated filamentation program.

To examine if Pum1 and Cfl1 represent the major targets of Znf2 in eliciting filamentation, we overexpressed *ZNF2* in the *cfl1*Δ, the *pum1*Δ, and the *pum1*Δ *cfl1*Δ mutants. The resulting overexpression strains were plated for single colonies under a mating-suppressive condition and were evaluated for the incidence of forming wrinkled colonies. The formation of wrinkled colonies reflects strong cell-cell adhesion and filamentation induced by the Znf2 controlled regulon [7]. The existence of a threshold level of Znf2 appears to control the colony morphological heterogeneity, with the whole population filamentous at a high expression level [7]. A population with a modest level of Znf2 displays a heterogeneous phenotype, likely reflecting the inherent fluctuation in the expression of Znf2's downstream genes in the population [7]. Similar phenomena are observed in another fungal pathogen *Candida albicans* in terms of the white-opaque switch controlled by the regulator Wor1 [35], [36], and in the bacterium *Bacillus subtilis* in terms of the motile-chained state switch controlled by the regulator SlrR [37]. In *Cryptococcus*, the overexpression of *ZNF2* in the wildtype strain evoked homogeneous wrinkled colony morphology in the population (Figure 4E), consistent with our previous study [7]. However, when *ZNF2* was overexpressed in the *pum1*Δ mutant or the *cfl1*Δ mutant, less than a third of the population showed wrinkled colony morphology. Double deletion of *PUM1* and *CFL1* almost abolished the ability of the population to form wrinkled colony morphology (<1%) in response to the *ZNF2* overexpression (Figure 4E). Thus, the fluctuation of the expression level of Pum1 and Clf1 likely contributes to the phenotypic heterogeneity in the population. Taken together, Pum1 and Cfl1 represent the major targets of Znf2 in directing filamentation and cell-cell adhesion in *C. neoformans*.

### Pum1 directs the initiation and the extension of hyphae partially through a secretory protein Fas1

**Figure 5:**
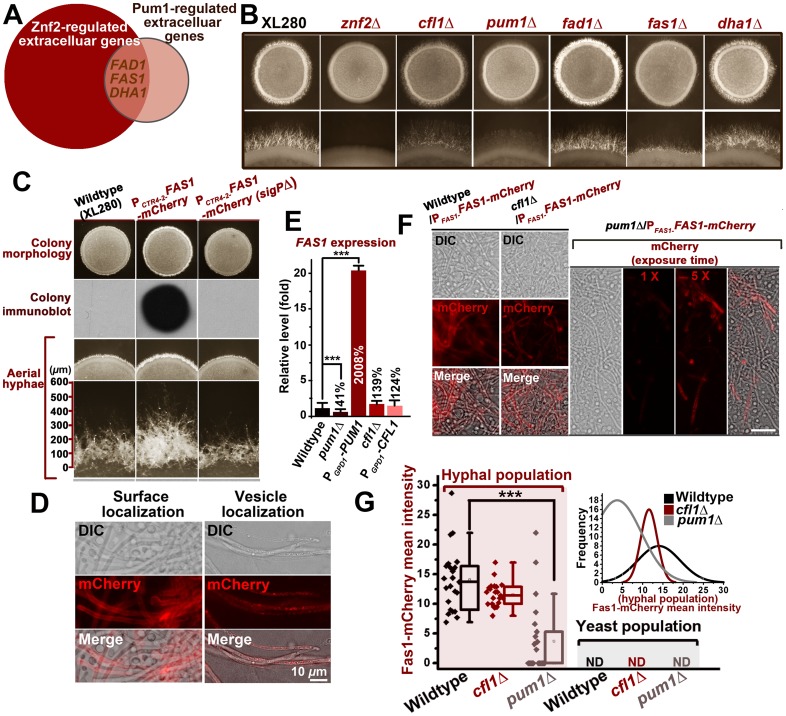
Pum1 directs hyphal initiation and extension partially through a novel hypha-specific extracellular protein Fas1. (A) *FAS1*, *FAD1*, and *DHA1* are the genes encoding extracellular proteins overlapped in the regulons of Znf2 and Pum1. (B) Fas1 recapitulates the role of Pum1 in unisexual hyphal production. The *fas1*Δ mutant, like the *pum1*Δ mutant, showed reduced abundance of aerial hyphae. (C) The indicated strains were grown on V8 agar plate containing 200 µM BCS in the dark at 22°C. Images of the colonies were photographed after 5 days of incubation. Released Fas1 proteins were detected by colony immune-blot. Only overexpression of Fas1 with intact signal peptide led to a more robust filamentation and longer hyphae. Thus, the secretion of Fas1 is crucial for its biological function. (D) Fas1-mCherry is observed on hyphal surface and in vesicles at 72 hrs post unisexual mating stimulation. (E) The *FAS1* expression level was positively regulated by Pum1 based on the transcriptional analysis of its transcript level in the *pum1*Δ mutant and the P*_GPD1_*-*PUM1* strain by qPCR. The deletion or the overexpression of *CFL1* did not significantly affect the *FAS1* expression level. (F and G) Pum1 increased the intensity of Fas1 expression and reduces its stochasticity. The average intensity of Fas1-mCherry fluorescence was dramatically reduced in the absence of Pum1, but not Cfl1. The deletion of *PUM1* led to a much higher variability in the Fas1-mCherry level among hyphal cells during unisexual filamentation (Inset of Figure? 5G). The frequencies of Fas1-mCherry's mean intensity in the hyphal population were plotted for the wildtype strain, the *cfl1*Δ mutant, and the *pum1*Δ mutant (software: ZEN 2011).

The results presented earlier highlighted the major roles of Pum1 and Cfl1 in directing hyphal morphogenesis. Next we wanted to examine if Pum1 and Cfl1 are functionally redundant or they have overlapping but distinct roles. A closer examination of the hyphal morphology in the absence of *CFL1* or *PUM1* suggests that Pum1 and Cfl1 have overlapping but distinct roles in hyphal growth. Although both mutants showed a reduction in the abundance of aerial hyphal production, hyphae produced in the *pum1*Δ mutant were notably shorter than that in the wildtype strain or the *cfl1*Δ mutant (Figures 4F and S4) during both bisexual and unisexual reproductions. Conversely, overexpression of Pum1 elicited a phenotype of longer hyphae compared with the wildtype (Figure S4). Thus Pum1 is not only important for hyphal initiation, but also for hyphal extension. Interestingly, overexpression of *ZNF2* could not bypass the defect of hyphal extension caused by the disruption of Pum1 (Figure S5), indicating that other Znf2 targets could not replace the role of Pum1 in controlling hyphal extension. To discover Pum1-mediated biological processes during hyphal development, we performed the whole-transcriptome microarray analysis of the hyper-filamentous *PUM1^OE^* strain. There were 85 genes exhibiting more than a 3 fold difference in response to *PUM1* overexpression (Table S2). In agreement with the RT-PCR result (Figure 4B), *CFL1* expression is not affected by the overexpression of *PUM1*. Gene ontology analyses indicated enrichment of two functional groups among the Pum1 regulon: one involved in meiosis and the other in extracellular proteins. The latter functional group (GO: 0005576) was also identified in the Znf2 regulon (Figure 1D). A comparison between the regulons of these two regulators revealed 3 genes encoding extracellular products [CNAG_00925 (named as *FAD1*), CNAG_05729 (named as *FAS1*) and Dha1] that were upregulated by overexpression of *ZNF2* and *PUM1* (Figure 5A). To evaluate their biological role in filamentation, we deleted these three genes. Only the deletion of CNAG_05729/*FAS1* impaired filamentation and recapitulated the phenotypes produced by the disruption of *PUM1* (Figure 5B). Conversely, overexpression of CNAG_05729/*FAS1* stimulated robust aerial morphogenesis and longer hyphal growth (Figure 5C), confirming its position as the major Pum1 target in quantitatively controlling various aspects of filamentation.

CNAG_05729 is predicted to encode a small secreted protein (154 aa) with a signal peptide for secretion at its N-terminus. The mCherry-fused protein was almost exclusively expressed in the filamentous subpopulation during mating colony differentiation (Figure 5D and F), which corroborates its involvement in the filamentation pathway. Thus, we named CNAG_05729 *FAS1* (Filament-Associated Secretory Protein 1). To investigate whether Fas1 is specially controlled by Pum1 or/and Cfl1, we assessed the impact on the expression of *FAS1* when *PUM1* or *CFL1* was either absent or overexpressed. Neither the absence nor the overexpression of *CFL1* altered the expression level of *FAS1*. By comparison, the transcript level of *FAS1* was positively correlated with that of *PUM1* (Figure 5E). At the protein level measured based on the fluorescence intensity of Fas1-mCherry, the deletion of *PUM1* led to a marked reduction in Fas1 expression in the hyphal subpopulation (Figure 5F). However, the level of Fas1-mCherry in hyphae was not significantly different in the presence or absence of *CFL1*, suggesting that Fas1 belongs to the Pum1 pathway but not the Cfl1 pathway. Interestingly, the variation in Fas1 expression among individual hyphae in the absence of Pum1 was noticeably higher, and some hyphal cells in the *pum1*Δ mutant did not produce any measurable Fas1 proteins (Figure 5F and G). This suggests that Pum1 may mediate a stochastic buffering-like regulatory mechanism [38].

To gain the clue for the molecular functions of Fas1, we employed a variety of bioinformatic tools to identify potential functional domains or motifs harbored in this small protein. However, no annotated domain was identified in Fas1. A PSI-BLAST analysis with regions of low complexity masked suggested that there are Fas1 homologues present in other fungal species, nearly all of them belong to the family of tremellaceae from the phylum of Basidiomycota. This suggests that Fas1 proteins may have arisen relatively recently. Most of the Fas1 homologues contain a signal peptide for secretion and are predicted to be secreted proteins.

We previously showed that the Basidiomycota-specific adhesion protein Cfl1 displays two patterns of subcellular localization: cell surface and intracellular vesicles that are morphologically similar in appearance to known fungal secretory vesicles [39], [40]. Similar localization patterns were also observed for Fas1-mCherry during hyphal development (Figure 5D). Like Cfl1, Fas1 can be also released into extracellular matrix, as released Fas1 from the community was easily detected by the colony immunoblot assay (Figure 5C). The deletion of the N-terminal signal peptide predicted for secretion abolished its secretion (Figure 5C). As expected, the ability of Fas1 to be secreted is critical for its function in enhancing filamentation and extending filamentous growth (Figure 5C).

### Pum1 bridges filamentation and meiosis during unisexual and bisexual mating

**Figure 6:**
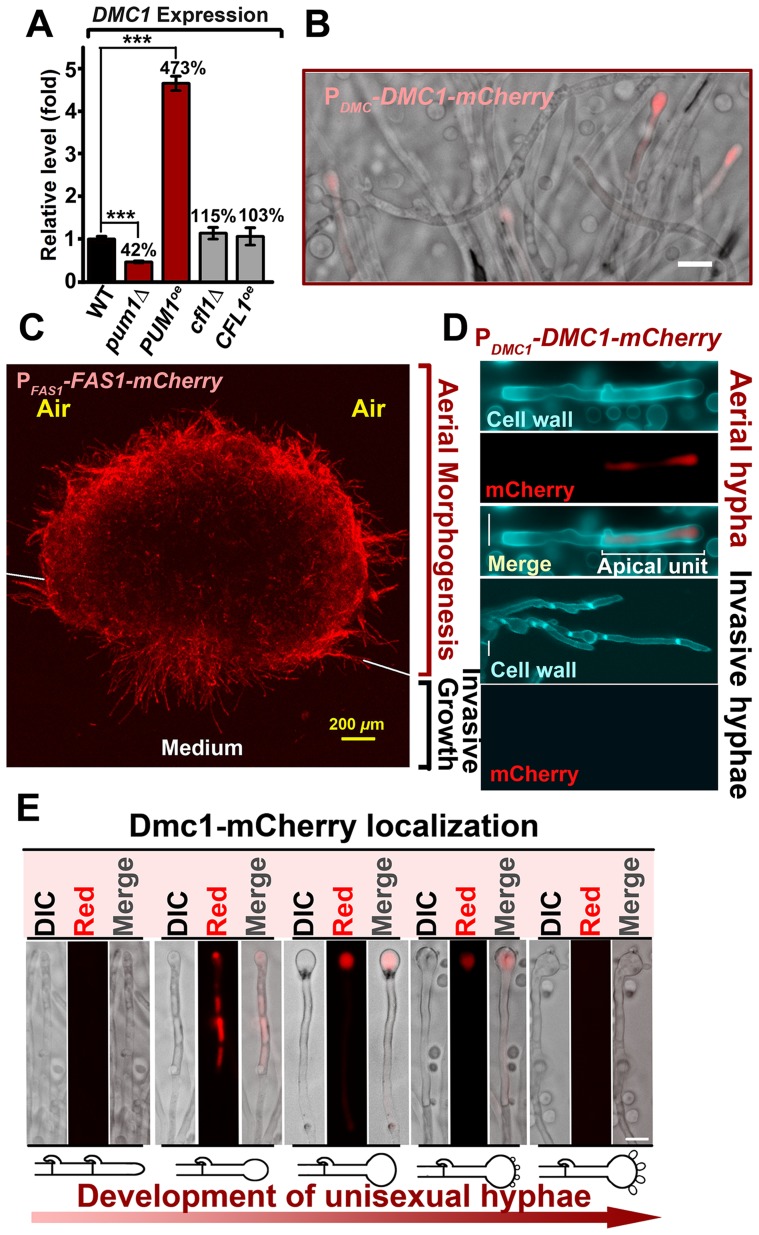
Unisexual meiotic progression is only observed in the Pum1-controlled aerial hyphal population. (A) Pum1, but not Cfl1, affects the expression of *DMC1* during unisexual reproduction. (B) Dmc1 expression is associated with a portion of hyphal cells but not yeast cells. Scale bar: 20* µ*m. (C) Fas1 is exclusively expressed in the hyphal population and it is used as a molecular indicator to visualize the hyphal population in a mating colony. A side-view of a single-cell derived colony (3 days old) shows the topology of Fas1-expressing hyphal population in the community. (D) Details of the morphology and Dmc1 expression for aerial hyphae and root hyphae (invasive hyphae) from the unisexual mating community are shown. Fungal cell wall was stained blue with calcofluor white. Dmc1 expression was exclusively observed in aerial hyphae but not in invasive hyphae (more than 200 invasive hyphae were examined). Scale bar: 10* µ*m. (E) The dynamics of Dmc1-mCherry monitored during unisexual development. Scale bar: 10* µ*m. The localization patterns of Dmc1 in the figure are representative for each given stage of development (>40 hyphae for each stage were examined).

The aforementioned results demonstrate the major roles of Pum1 and Cfl1 in directing aerial hyphal morphogenesis, a process that could lead to the formation of fruiting bodies (sexual sporulation) critical for species survival and expansion into new niches. The enrichment of meiosis and sporulation genes in the Pum1 regulon in addition to the filamentation genes (e.g. *FAS*1) raises the possibility that Pum1 may bridge the progression from filamentation to meiosis and to post-meiotic sporulation during sexual reproduction. One meiosis-specific gene that was significantly upregulated in the *PUM1^OE^* strain is *DMC1*. Dmc1 is the recombinase specifically involved in homologous recombination at an early stage of meiosis. Earlier investigations have demonstrated that *DMC1* is important for the formation of tetrad spore chains in *Cryptococcus* [12], [16]. Overexpression of *PUM1* resulted in a greater than 4 fold increase in the *DMC1* transcript level whereas the deletion of *PUM1* led to a two-fold reduction in its expression (Figure 6A). By contrast, we did not observe any significant impact on *DMC1* expression by altering the expression level of *CFL1* (Figure 6A). The results reinforce the notion that Pum1 and Cfl1 are functionally different and support our hypothesis that Pum1 may control subsequent development following filamentation through its regulation of factors such as *DMC1*.

**Figure 7:**
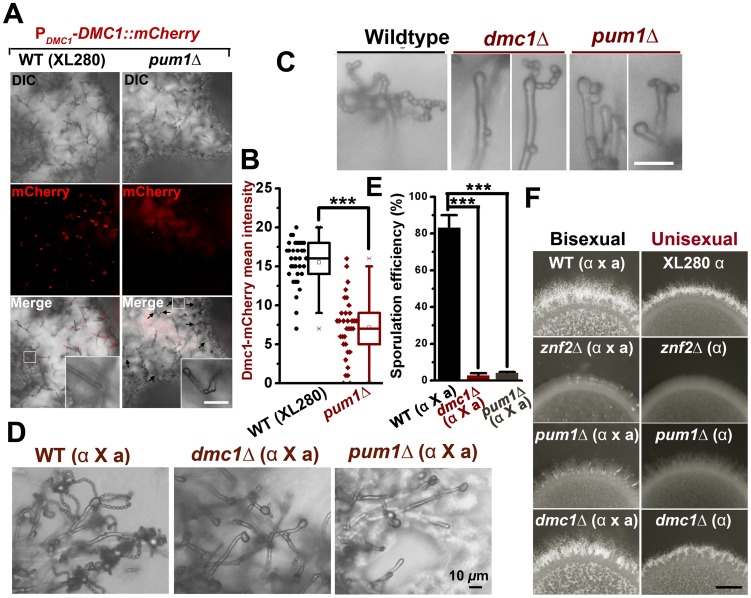
Pum1 coordinates filamentation and sexual reproduction. (A and B) The deletion of *PUM1* impairs the expression of Dmc1 in basidia in a stochastic manner. Some but not all differentiating basidial heads lost the expression of Dmc1-mCherry without Pum1. Those that did expressed Dmc1at lower levels. Scale bars: 20* µ*m. (C) The unisexual sporulation was perturbed in the absence of the meiosis-specific protein Dmc1 and the global regulator Pum1. The wildtype strain XL280, the *dmc1*Δ mutant, and the *pum1*Δ mutant were diluted on V8 agar to form single colonies. Typical mature basidia observed from these strains were shown. Scale bar: 10* µ*m. (D and E) The deletion of *PUM1* and *DMC1* also dramatically reduced the sporulation efficiency during bisexual mating. (F) The effect of the deletion of *ZNF2*, *PUM1*, or *DMC1* on aerial hyphal morphogenesis at the colony level during both bisexual and unisexual development. Scale bar: 1 mm.

The function of Dmc1 in the progression of meiosis is extremely conserved among evolutionally divergent eukaryotes [41] and it serves as a molecular marker for the meiotic process [42]. Therefore, we decided to use Dmc1-mCherry controlled by the *DMC1*'s native promoter to identify the cell type associated with Dmc1 and monitor meiotic progression during the development of a unisexual mating community. Dmc1-mCherry was only detected in apical cells in a portion of hyphae and undetectable in yeast cells (Figure 6B and D). To further specify the subpopulation of hyphae associated with Dmc1 expression, we probed the topological pattern of the hypha-specific protein Fas1 in a unisexual community derived from a single cell using confocal microscopy (Figure 6C). The vertical transverse cross section of the colony indicated that the aerial hyphal population was enriched in the peripheral region of the upper colony while invasive filaments radially rooted the colony into the agar (Figure 6C). Interestingly, Dmc1-mCherry was detected only in some of the aerial hyphae, and not in any of the invasive hyphae (Figure 6B and D). A closer examination of aerial hyphae showed that Dmc1-mCherry was not detected in actively growing hyphae with no sign of differentiation into basidia. Rather, an intense signal was detected in the apical compartment of aerial hyphae when their apexes began to differentiate into basidia (Figure 6E). The signal gradually became focused in the basidial head during basidial maturation until four pre-spores started to emerge. The signal disappeared from the basidia when spores matured (Figure 6E). The dynamic expression of Dmc1 mirrors the progression from vegetative aerial hyphal growth to unisexual meiosis and sporulation. Given that mutations of *PUM1* affect the transcript level of *DMC1* (Figure 6A), we decided to examine the impact of the deletion of *PUM1*on Dmc1 protein expression. Remarkably, many aerial hyphal apexes differentiating into basidia lost the signal of Dmc1-mCherry in the *pum1*Δ mutant during unisexual colony development (Figure 7A). For those that had the Dmc1-mCherry signal, the fluorescent intensity was significantly lower (Figure 7B). Again, the loss of Pum1 increased the stochasticity of Dmc1 dynamics in basidia (Figure 7B) as observed for Fas1 in filaments (Figure 5G).

Previous studies indicated that mutations of meiosis-specific genes perturb sporulation [12], [16]. The effect of such mutations are highly specific, as the deletion of *DMC1* or *SPO11*(an endonuclease creating double-stranded breaks to initiate meiosis) did not affect hyphal initiation, hyphal extension, or the terminal differentiation of some aerial hyphae into basidia (Figure 7F)[12]. The deletion of *PUM1* likewise resulted in a severe defect in the formation of basidia and appropriate tetrads of spore chains during either unisexual or bisexual reproduction (Figure 7C, D, E and F). However, disruption of Pum1 also drastically reduced the ability of this fungus to initiate and to maintain hyphal growth (Figures 2B and 4F). These results suggest that Pum1 links sexual reproduction with filamentation, and meiosis *per se* does not influence earlier morphogenesis events. Although most of the studies were performed in a laboratory strain XL280 background, we confirmed that Pum1 plays a similar role in bridging sex and filamentation in a clinical isolate H99 (Figure S6). Our data presented here indicate that Pum1 plays a conserved role in genetically orchestrating the transition from yeasts to hyphae and the subsequent transition from aerial hyphae to sexual reproduction in this eukaryotic pathogen.

## Discussion

Facing unpredictable changes in the environment, terrestrial microbes have evolved sophisticated adaptation strategies. One well-known example is morphotype transition [3], [6], [10], which essentially achieves a functional transition by changing cell shape or size [3]. This strategy is commonly adopted by environmental pathogens to survive in the environment and inside a host, conditions that could differ drastically from each other [3], [6]. As the transition between yeasts and hyphae is linked to pathogenesis in many environmental fungal pathogens [3], [6], [43], [44], [45], and that morphogenesis is an integral part of fungal development, understanding the molecular mechanisms controlling fungal morphogenesis is crucial in our understanding of fungal pathogenic strategies and fungal biology. Because morphotype transition in *Cryptococcus* is a stochastic process, identification of phase-specific genes and their regulation systems in this organism has been challenging.

In this study, we took advantage of an engineered cryptococcal strain to generate homogeneous yeast and hyphal populations. This approach enabled a more sensitive comparison between the two cryptococcal morphotypes and allowed us to uncover a more reliable Znf2 regulon. Functional classification of this regulon revealed similar genetic programs related to filamentous growth controlled by Znf2 in *Cryptococcus* as the ones regulated by Rbf1 in the basidiomycete plant pathogen *Ustilago maydis* [46], [47]. Although Rbf1 is much smaller than Znf2, both regulators contain the similar C2-H2 Zinc-finger DNA binding domain [47] (data not shown). A considerable proportion of Rbf1's regulon also encode secretory proteins and factors involved in cell cycle progression [47], [48]. Such a striking parallelism in *Cryptococcus* and *Ustilago* suggests that Znf2 and Rbf1 might have evolved from a common regulator, which controlled the morphological transition in an ancient Basidiomycota species.

The inherent properties of yeasts or hyphae might dictate the necessary genetic programs common in diverse fungi regardless of the upstream species-specific regulatory systems. For instance, extracellular proteins and cell wall-modifying proteins are required to reconstruct different cell shapes [30], [32]. Cell cycle progression is overrepresented in a yeast population compared to a filamentous population [30] likely because all but the apical compartments are quiescent in the hyphal population. Given that the yeast-to-hypha transition is observed in major fungal phyla [3], [5], [30], [32], [49], the ability to undergo morphological switches might have existed in original fungi prior to the demarcation of these phyla. Species-specific features could be wired later into the regulation of morphological transition to optimize the adaptation of each fungal species to their unique natural niches, as in the case of mating-initiated yeast-hyphal transition in *C. neoformans* and in *U. maydis*. In both organisms, filamentation is developmentally associated with the progression of sex, which helps ensure species' long term success through the creation of genetic variants and infectious spores [22], [23], [24], [50], [51].

**Figure 8:**
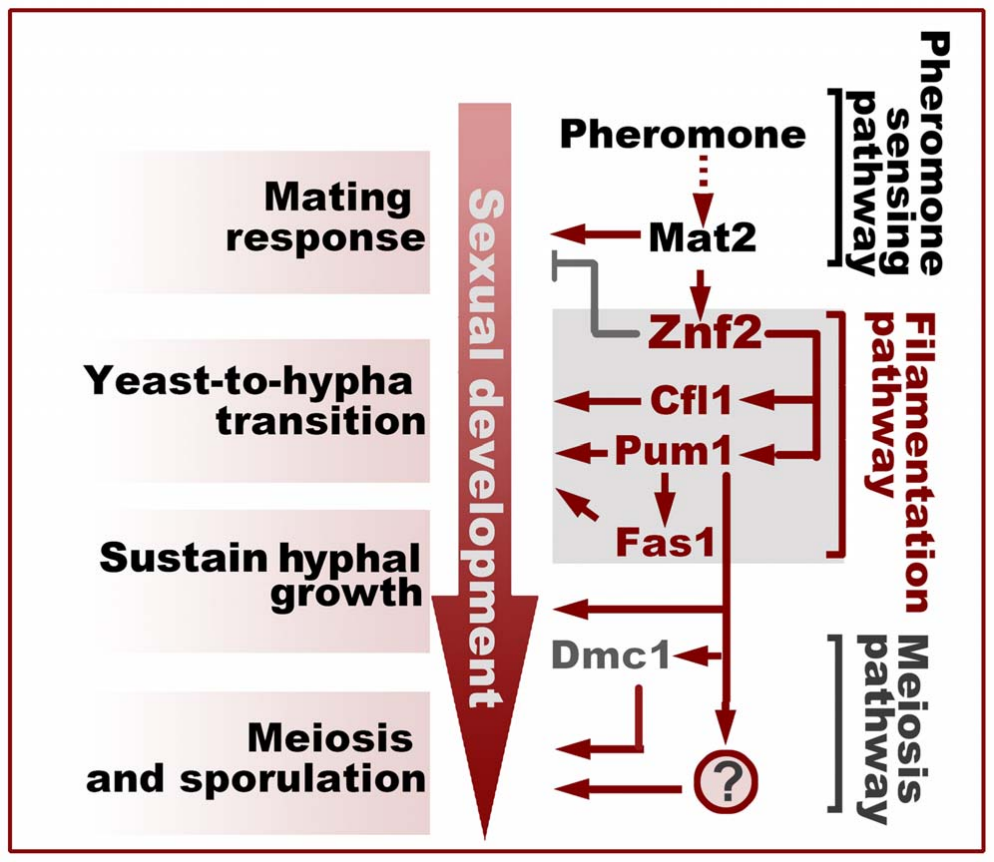
Filamentation pathway coordinates sexual reproduction and morphogenesis in *Cryptococcus neoformans*. Activation of Znf2 promotes the filamentous growth and modestly inhibits the early mating behaviors that initiate the transition from the yeast form to the hypha form [7], [12]. Cfl1 and Pum1 represent two parallel major branches downstream of Znf2 in the orchestration of this morphological switch. Pum1 plays additional important roles in sustaining hyphal growth and in bridging hyphal development and sexual reproduction partially through its control over the expression of *DMC1* and *FAS1*. The question mark indicates unidentified factors controlled by Pum1 that regulate meiosis and sporulation.

In the development of a cryptococcal mating community, the yeast-to-hypha transition is followed by sustained hyphal growth and subsequent formation of fruiting bodies from aerial hypha apexes. Although it is clear that Znf2 controls filamentation in *Cryptococcus* and a few proteins like Dmc1 and Spo11 are involved in meiosis, the factor that connects filamentation with sexual reproduction remains elusive (Figure 8). From the Znf2 regulon, we found two major regulatory branches downstream of Znf2 controlling the yeast-to-hypha transition: Pum1 and the matricellular signal Cfl1. Cfl1 does not play a major role in promoting sustained hyphal growth or the fruiting body formation, suggesting that this signal probably exerts a dedicated control at the stage of hyphal initiation [2] (Figure 8). By contrast, Pum1 is important for multiple developmental stages. Pum1 promotes yeast-to-hypha transition in a mating colony through its regulation of hypha-specific proteins (e.g. Fas1) (Figures 5F and 8); Pum1 bridges the progression from aerial hyphal growth to meiosis through its additional regulation on multiple meiosis-related genes (Figure 8). The representation of genes involved in diverse functions in the Pum1 regulon (Table S2) is also consistent with its pleiotropic role. One of the interesting finding of this study is the dynamic expression pattern of the meiosis-specific recombinase Dmc1. Dmc1 is undetectable in undifferentiating growing hyphae (none in invasive hyphae), and is only expressed in aerial hypha apexes that are differentiating into fruiting bodies (Figures 6B and 7A). The concurrence of the onset of meiosis and the terminal differentiation of hyphal growth represents a culminating feature for the committed sexual reproduction. The dynamics of the expression of the highly conserved meiosis-specific protein during the development of basidia (Figure 6E) in a unisex colony provides yet another strong piece of evidence for the meiotic nature of this novel life cycle [16].

What remains to be established is the mode of action of Pum1. Pum1 contains an mRNA-binding Pumilio domain. The members of this family control a variety of biological processes through modulating the stability and the translation of their target transcripts [33]. Pumilio proteins are generally considered repressors and they recruit the deadenylase to decay their target mRNAs [33]. However, accumulating evidence indicates that regulators of this family can also function as activators [33]. One such example is Puf9 from the parasite *Trypanosoma brucei* [52]. Puf9 in *T. brucei* stabilize its mRNA targets, likely through a competition with a repressor for the mRNA binding [52]. Here, we show that Pum1 also plays a positive role in the control of the gene expression (e.g. *FAS1* and *DMC1*). Loss of Pum1 decreases the overall expression levels of *FAS1* and *DMC1* but increases the variations of these protein expression levels among cells in the same population. This suggests that Pum1 may mediate a stochastic buffering-like regulatory mechanism [38]. It remains to be determined if Pum1 positively controls the expression level of its targets through promoting their transcription, stabilizing their mRNAs, or facilitating their translation. Further investigations into Pum1-interating factors and the dynamics of target mRNAs at different developmental stages would help obtain a mechanistic understanding of how Pum1 coordinates hyphal and sexual development.

## Materials and Methods

### Ethics statement

All the animal experiments were performed according to the guidelines of NIH and Texas A&M University Institutional Animal Care and Use Committee (IACUC permit # 2011–22).

### Statistics

The programs Originpro and GraphPad Prism were used for statistical analyses in this study. A two-tailed unpaired Student *t* test was performed to compare the mean florescence intensity or transcript levels from two groups. *P* values less than 0.05 were considered statistically significant.

### Strains and growth conditions

Strains used in this study are listed in Table S3. For both unisexual and bisexual mating assays, strains were maintained on V8 agar unless indicated otherwise. The optical density of cell suspensions measured at 600 nm was used to represent cell density. When the inducible promoter of the copper transporter gene *CTR4* (e.g. P*_CTR4-2_*-*FAS1*) was used to drive the genes, the gene expression level was manipulated by the addition of 25 µM CuSO_4_ (inhibitor) or 200 µM the copper chelator Bathocuproine disulphonate (BCS; inducer) to the media as described previously [7], [53], [54].

### UV mutagenesis and suppressor screen for filamentation mutant

Overnight culture of the *znf2*Δ mutant in YPD liquid medium was plated on YPD agar. The plates were dried and then explored to UV light (300 J/m^2^) for 3 seconds by using the UV cross linker. The cells were then collected, washed by PBS and plated on V8 agar medium for 7 days in the dark at 22°C to form visible colonies. The colonies were screen under a stereoscope for the formation of hyphae. The mutants with hypha-like morphology were furthered examined with a compound microscope. No true hyphae were observed in any of the mutants screened but mutants with pseudohyphae and/or shmoo cells were observed.

### Mating and self-filamentation assays

For bisexual mating assays, **a** and α cells of equal number (original optical density of 1.5 at OD_600_) were cocultured on V8 juice agar medium (pH = 7.0 for serotype D strains, pH = 5.0 for serotype A strains) in the dark at 22°C [55]. Random basidiospores were isolated using a micromanipulator. For unisexual mating assay (serotype D XL280 strain background), cells were spotted on V8 medium alone. Both bisexual and unisexual matings were examined microscopically for formation of mating hyphae and chains of basidiospores.

### Gene knockout and overexpression

Primers used in this study are listed in Table S4. To disrupt the *PUM1*, *FAS1, FAD1, DHA1* or the *ZNF2* genes, an overlap PCR products were generated with the NAT or the NEO dominant drug marker amplified from plasmid pAI1 or pJAF1 [11] and 5′ and 3′ flanking sequences (1∼1.5 kb) of the coding genes from strain JEC21α or H99α as we described previously [11]. The PCR product was directly introduced into strains JEC21α, XL280α, JEC20**a**, or H99α by biolistic or electronic transformation. The resulting deletion mutants generated *via* homologous recombination were confirmed by PCR and genetic crosses. For overexpression, an overlap PCR product with the NEO or NAT marker and the wild-type genes containing their native promoter (∼1.2 kb) and *GPD1* terminator (∼0.5 kb) from strain JEC21 or H99 was generated. The PCR products were directly introduced into appropriate backgrounds as indicated in the text. Mutant strains in the *MAT* **a** background were obtained by crossing the corresponding α mutants with the widltype congenic **a** strains. For overexpression, genes (ORF) were amplified by PCR and the amplified fragments were digested and inserted into pXL1 or pXC after the *GPD1* or the *CTR4-2* promoters respectively as we previously described [7]. Overexpression plasmids were linearized *via* restriction enzyme digestion before being introduced into the relevant *Cryptococcus* strains as we previously described [7].

### Murine models of cryptococcosis

Animals were intranasally infected as previously described [7], [56], [57]. Nine A/J mice (6- to 8-week-old female; Jackson Labs) per group were used for survival studies. Statistical significance (*P*≤0.05) of the survival data between different groups was assessed by the Mantel-Cox log-rank test [58].

### Cell fusion assay

The coculture of α and **a** cells with different auxotrophic markers (*lys* for α; *ade2* for **a**) were inoculated on V8 agar or in YPD liquid medium. After 22 hrs of incubation, the coculture was collected, washed by cold water twice, and plated on minimum YNB agar to select fusion products at 37°C as described previously [11].

### Construction of fluorescent proteins and microscopic examination

The mCherry was fused to the C-terminus of the interested protein used in this study. Overlap PCR was used to piece together the fragments including the coding region of the gene with 1∼1.2 kb upstream sequences and the mCherry. The resulting products were introduced into plasmid pXL1 as described previously [7]. The *FAS1-mCherry* without the promoter was amplified and introduced to pXC to generate the plasmid P*_CTR4-2_-FAS1-mCherry*. To overexpress Fas1-mCherry that lacks the N-terminal signal peptide [Fas1 (sigPΔ)-mCherry], primers Linlab 1581 and Linlab864 were used to generate *FAS1(sigP*Δ*)-mCherry* allele. The P*_CTR4-2_*-*FAS1*-mCherry construct was used as the template. The resulting PCR product was cloned into pXC to produce P*_CTR4-2_*-*FAS1*(*sigP*Δ)-*mCherry*. To examine the sub-cellular localization of morphogenesis or meiosis-related proteins during filamentation, the fluorescence strains were grown on V8 agar medium at 22°C for 72 hrs before being examined microscopically. Images were acquired and processed with a Zeiss Axioplan 2 imaging system with the AxioCam MRm camera software Zen 11 (Carl Zeiss Microscopy). To assess the expression level of P*_CTR4-2_* -*FAS1*-mCherry and P*_CTR4-2_*-*FAS1*(*sigP*Δ)-mCherry in a community, the overexpression strains were grown on V8 agar medium containing the copper chelator BCS before examined with the fluorescence stereoscope.

### RNA purification, qPCR analyses, and microarray analyses

The purelink RNA kit (Invitrogen) and the Superscript III cDNA synthesis kit (Invitrogen) were used for total RNA purification and the first strand cDNA synthesis respectively according to the manufacture instruction. The house-keeping gene *TEF1* was used as the endogenous control for normalizing gene expression levels. The relative transcript levels were determined using the comparative CT method as described previously [7].

For transcriptional profiling of different morphotypes, the yeast H99 wildtype strain and the filamentous P*_CTR4-2_-ZNF2* strain were grown in YPD liquid medium containing BCS at 22°C for 48 hrs. The cell morphology and the proportion of hyphal and yeast populations were measured before the total RNAs were extracted. RNAs were processed for microarray analyses as described previously [7]. For the transcript analysis to identify the Pum1 regulon using microarray, XL280 and the P*_GPD1_-PUM1* strain were grown on V8 agar medium at 22°C for 72 hrs. Unisexual filamentation and sporulation were confirmed microscopically before extraction of total RNAs. The arrays were analyzed similarly as described previously [11].

### Protein extraction and western-blot analyses

For Western Blot analysis of the Cfl1 expression, overnight cultures of indicated strains in YPD liquid medium were spotted onto V8 agar for 3 days at 22°C. The cells were removed and suspended in cold PBS for protein extraction. Protein extraction and western analysis were performed as described previously [2]. Briefly, the cell suspension was subjected to centrifuging at 13,000xg for 10 min at 4°C. The pellet was freeze dried and disrupted. The disrupted cells were subsequently suspended for western immunoblotting analysis of whole-cell proteins. The house keeping protein β-actin was used as the endogenous control.

### Colony immunoblot for the detection of secreted Fas1 and Cfl1 proteins

For colony immunoblot assays, 3 microliters of cell suspension with the density of 1.5×10^7^ cells/mL were spotted onto the same plate for the assay as described previously [2]. The strains were grown on V8 agar medium containing BCS at 22°C for 24 hrs to form visible colonies and the plate with the colonies was laid over by a sterile nitrocellulose membrane (Millipore, Billerica, MA). The membrane was removed from the plate after incubation for additional 3 days. The membrane was subsequently washed with 1× Tris-buffered saline (TBS) to remove attached cells. The blots were incubated with the anti-mCherry primary antibody (1/1000 dilution) and then a rabbit anti-mouse secondary antibody (1/10,000 dilution). Detection was performed using the ECL system according to the manufacture's instruction.

### Confocal microscope analyses

P*_DMC1_-DMC1-mCherry* or P*_FAS1_-FAS1-mCherry* were grown on V8 (pH7.0) plates for 5 days at room temperature in dark to form visible colonies. For probing *DMC1* expression in different hyphal subpopulations, 50 µg/ml calcofluor white (stain cell wall) was added into the medium to enable the visualization of all fungal cell types. For sample preparation, colonies were embedded in low-gelling agarose (sigma-Aldrich) on the plates [59]. Immediately after gelling, agarose-embedded colonies were cut vertically in the middle and transferred to the cover glass with the longitudinal section facing the cover glass for the side view of the colonies. Cover the sample with 1∶9 PBS-Glycerol. An Olympus Fv1000 confocal laser scanning microscope and an Olympus IX81 spinning disk confocal microscope were used for the image acquisition. For Olympus Fv1000 confocal laser scanning microscope, calcoflour white and mCherry were excited with 488 nm argon and 543 nm He-Ne laser lines respectively using a dry 40× objective. For Olympus IX81 spinning disk confocal microscope, calcoflour white and mCherry were excited with 405 nm argon and 561 nm laser lines respectively with dry 40× objective. The data analyses were carried out with MetaMorph Microscopy Automation & Image Analysis Software (Molecular Devices, PA).

## Supporting Information

Figure S1 **Overexpression of** ***ZNF2*** **yields a homogenous hyphal population.** Images above show the homogenous yeast population, hyphal population, and the heterogeneous population. These populations were generated by the wild type strain without mating stimulation, the P*_CTR4-_2-ZNF2* strain in the presence of BCS, and the wild type strain with mating stimulation respectively. The histogram below depicts the difference in two transcriptome analyses. The current approach (red) used population with a homogeneous morphotype and yielded a higher number of genes with significantly differential expression between the yeast and filamentous growth. The previous approach (black) used the wildtype population in response to mating stimulation. The population showed pronounced heterogeneity in cell morphotype and yielded a much smaller number of phase-specific genes.
(TIF)

Figure S2 ***PUM1*** **is a downstream target of Znf2.** (A) A schematic diagram of predicted domains of Pum1. Pum1 is a member of the Pumilio regulator family. It contains the PUM-HD (Pumilio-Homology Domain), which is generally involved in gene regulation *via* binding to RNAs. (B) The *PUM1* expression level was positively regulated by Znf2 based on the transcriptional analysis by qPCR. The expression level of the *PUM1* gene in the wildtype strain XL280 was arbitrarily set as 1 for comparison.
(TIF)

Figure S3 **Cfl1 and Pum1 are not engaged in invasive growth under different conditions.** (A) The deletion of *PUM1* or *CFL1* drastically reduced the abundance of aerial hyphae (white fluffy appearance of the colony) but not invasive growth during bisexual mating on V8 agar medium. In comparison, the deletion of *ZNF2* abolished hyphal growth and invasive growth. (B) The indicated strain pairs were mixed and incubated on YPD agar medium (mating-suppressive) for 5 days before being photographed. The deletion of *ZNF2*, but not *PUM1* or *CFL1* abolished invasive growth. Scale bar: 2 mm.
(TIF)

Figure S4 **Pum1 but not Cfl1 is involved in promoting hyphal extension during unisexual reproduction.** The strains with same cell concentration were spotted onto V8 agar and incubated for 5 days. Hyphal length was positively related to the *PUM1* expression level (disrupted or overexpressed). Cfl1 did not have any major impact on hyphal extension during unisexual mating. Scale bars: 30* µ*m.
(TIF)

Figure S5 **The overexpression of** ***ZNF2*** **cannot bypass the requirement for** ***PUM1*** **in promoting sustained hyphal growth.** The aerial hyphae were extremely sparse in the *pum1*Δ/*cfl1*Δ mutant even in response to *ZNF2* overexpression, which prohibited further analysis of aerial hyphal length in this strain. By contrast, invasive hyphae were only modestly affected when *ZNF2* was overexpressed in this double deletion mutant. The *ZNF2* overexpression did not restore the defect in hyphal extension in the absence of Pum1.
(TIF)

Figure S6 **Pum1 orchestrates filamentation and sporulation in the clinical isolate H99 during bisexual mating.** (A) Deletion of *PUM1* impaired filamentation and abolished sporulation during bisexual mating in the H99 background. (B) Pum1 has a minor role in controlling invasive growth but is required for hyphal extension.
(TIF)

Table S1 **Genes differentially expressed in response to the** ***ZNF2*** **overexpression under the mating-suppressing condition.**
(XLS)

Table S2 **Genes differentially expressed in response to the** ***PUM1*** **overexpression under the mating-inducing condition.**
(XLS)

Table S3 **Strains used in this study.**
(DOC)

Table S4 **Primers used in this study.**
(DOC)
