# Supplementary figures and images for: Morphotype Transition and Sexual Reproduction Are Genetically Associated in a Ubiquitous Environmental Pathogen
Source: PLoS Pathog. 2014 Jun 5;10(6):e1004185. doi: 10.1371/journal.ppat.1004185 (PMC4047104; doi:10.1371/journal.ppat.1004185)

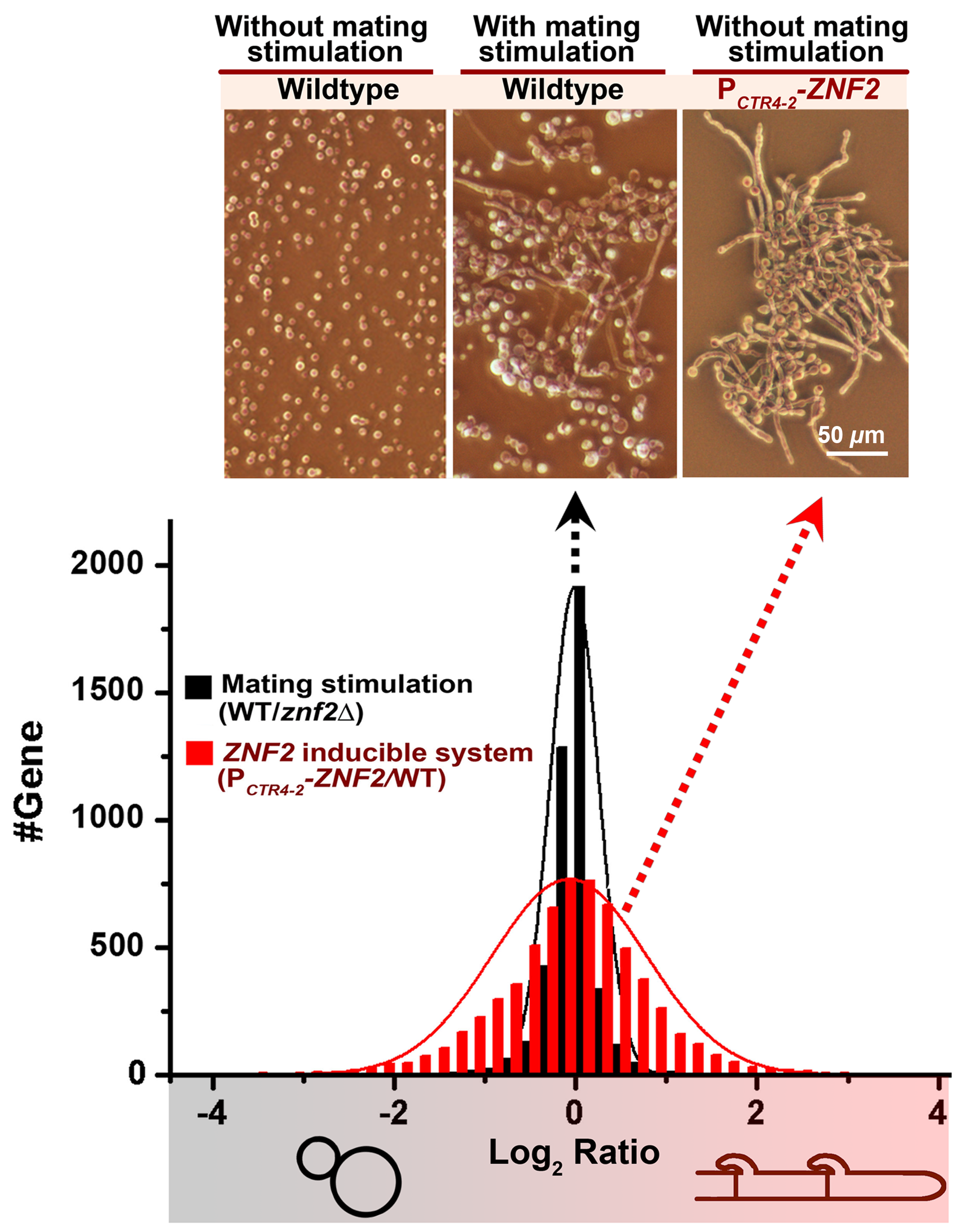

Supplement: Figure S1 — Overexpression of ZNF2 yields a homogenous hyphal population. Images above show the homogenous yeast population, hyphal population, and the heterogeneous population. These populations were generated by the wild type strain without mating stimulation, the PCTR4-2-ZNF2 strain in the presence of BCS, and the wild type strain with mating stimulation respectively. The histogram below depicts the difference in two transcriptome analyses. The current approach (red) used population with a homogeneous morphotype and yielded a higher number of genes with significantly differential expression between the yeast and filamentous growth. The previous approach (black) used the wildtype population in response to mating stimulation. The population showed pronounced heterogeneity in cell morphotype and yielded a much smaller number of phase-specific genes. (TIF) [file ppat.1004185.s001.tif]

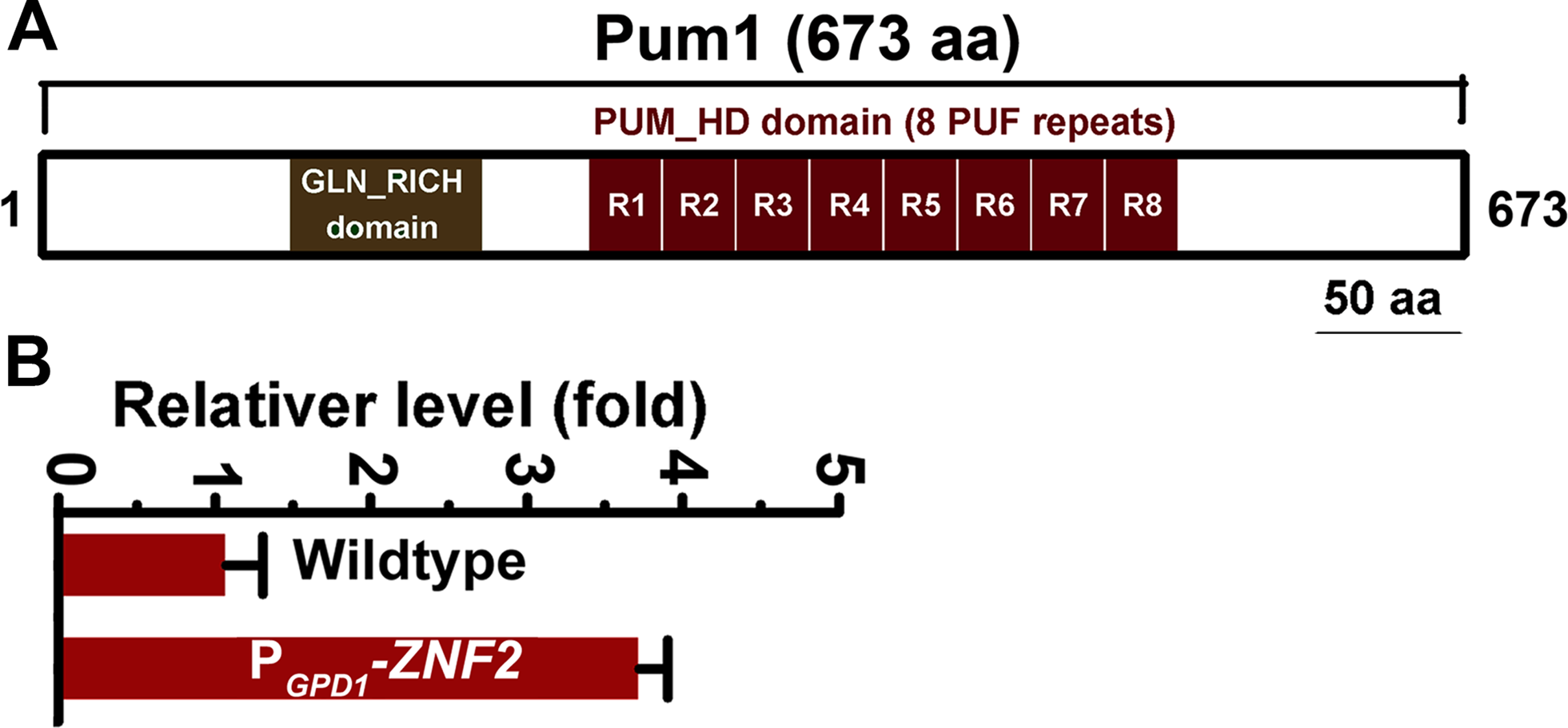

Supplement: Figure S2 — PUM1 is a downstream target of Znf2. (A) A schematic diagram of predicted domains of Pum1. Pum1 is a member of the Pumilio regulator family. It contains the PUM-HD (Pumilio-Homology Domain), which is generally involved in gene regulation via binding to RNAs. (B) The PUM1 expression level was positively regulated by Znf2 based on the transcriptional analysis by qPCR. The expression level of the PUM1 gene in the wildtype strain XL280 was arbitrarily set as 1 for comparison. (TIF) [file ppat.1004185.s002.tif]

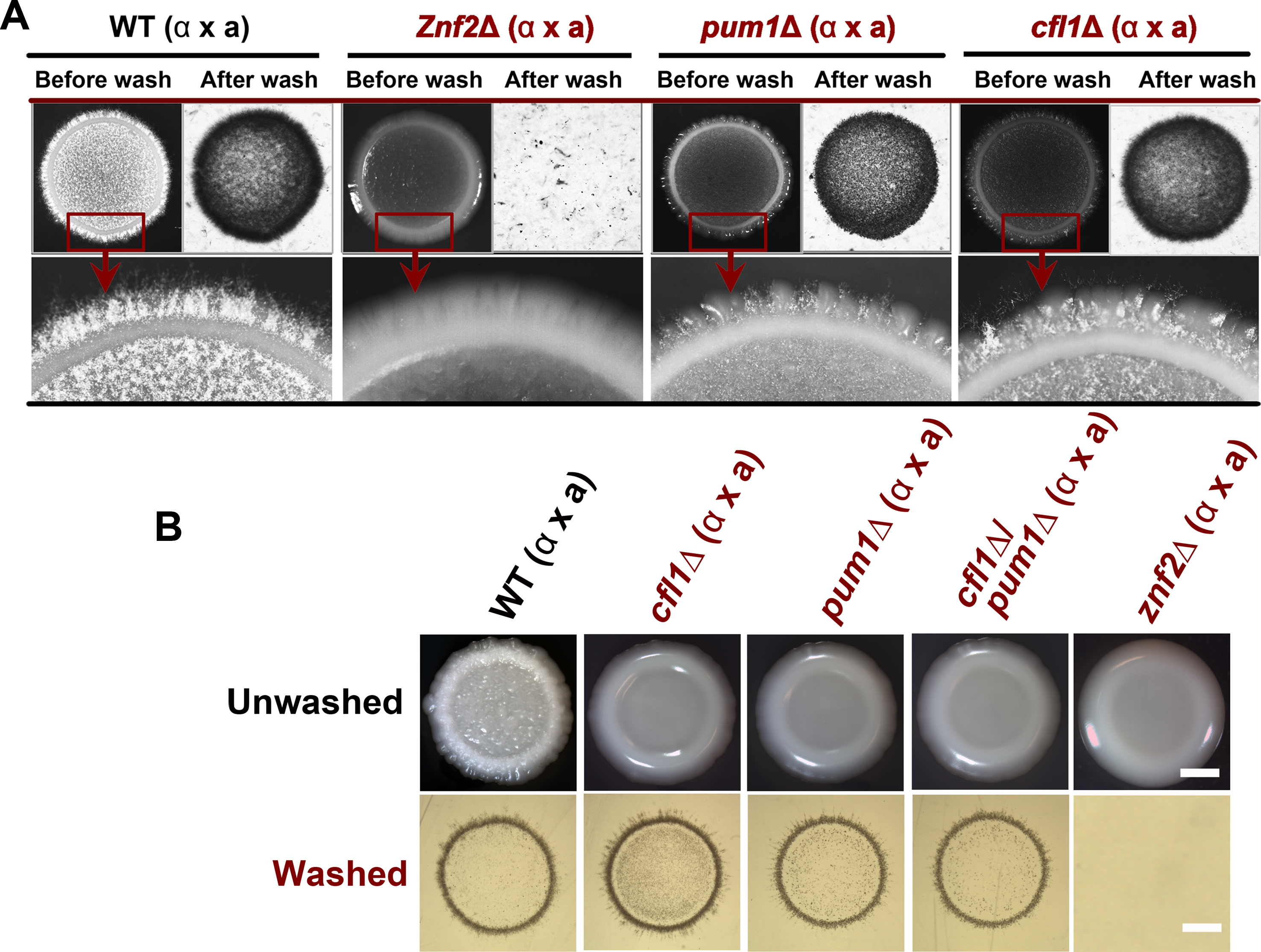

Supplement: Figure S3 — Cfl1 and Pum1 are not engaged in invasive growth under different conditions. (A) The deletion of PUM1 or CFL1 drastically reduced the abundance of aerial hyphae (white fluffy appearance of the colony) but not invasive growth during bisexual mating on V8 agar medium. In comparison, the deletion of ZNF2 abolished hyphal growth and invasive growth. (B) The indicated strain pairs were mixed and incubated on YPD agar medium (mating-suppressive) for 5 days before being photographed. The deletion of ZNF2, but not PUM1 or CFL1 abolished invasive growth. Scale bar: 2 mm. (TIF) [file ppat.1004185.s003.tif]

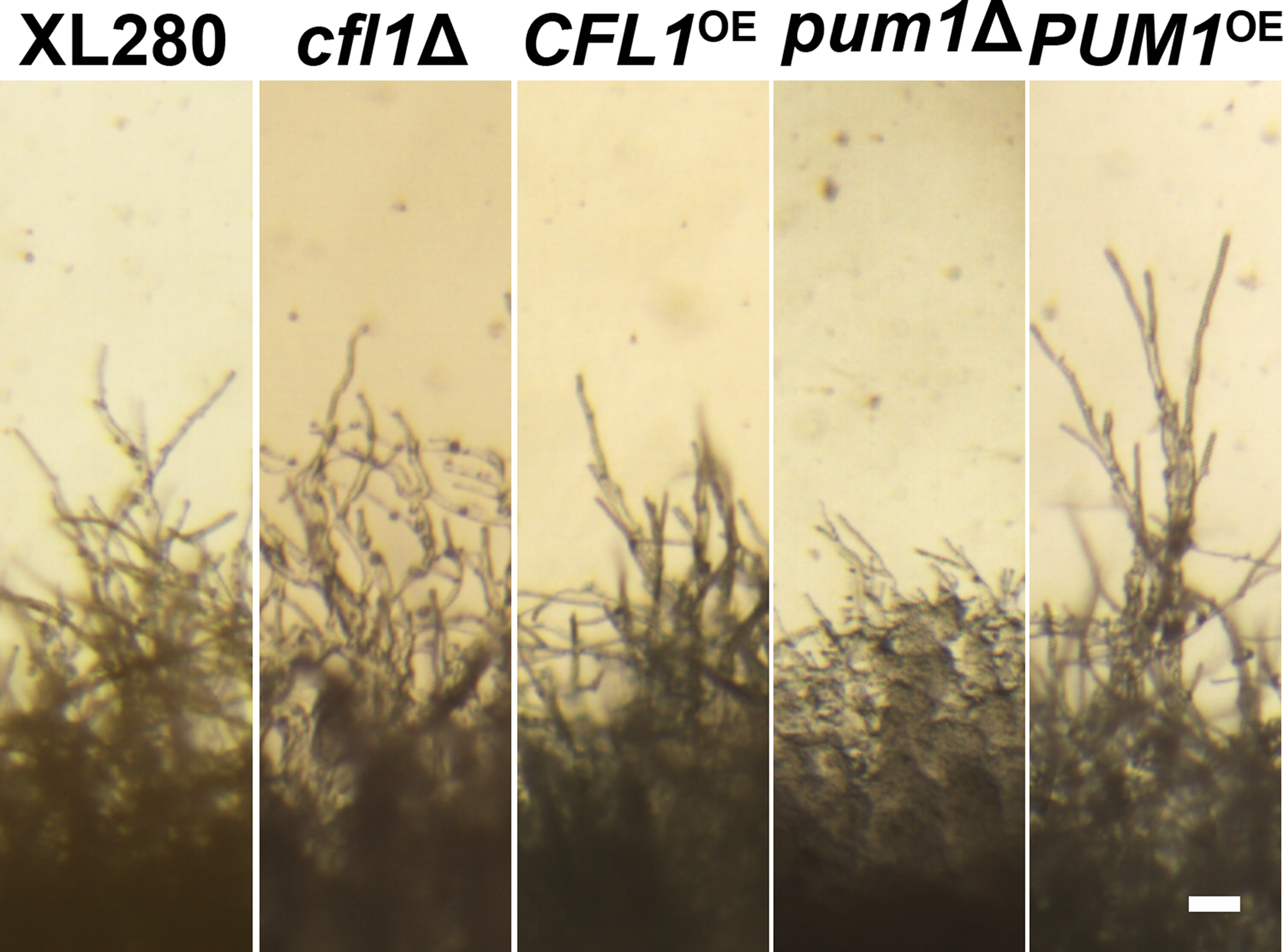

Supplement: Figure S4 — Pum1 but not Cfl1 is involved in promoting hyphal extension during unisexual reproduction. The strains with same cell concentration were spotted onto V8 agar and incubated for 5 days. Hyphal length was positively related to the PUM1 expression level (disrupted or overexpressed). Cfl1 did not have any major impact on hyphal extension during unisexual mating. Scale bars: 30 µm. (TIF) [file ppat.1004185.s004.tif]

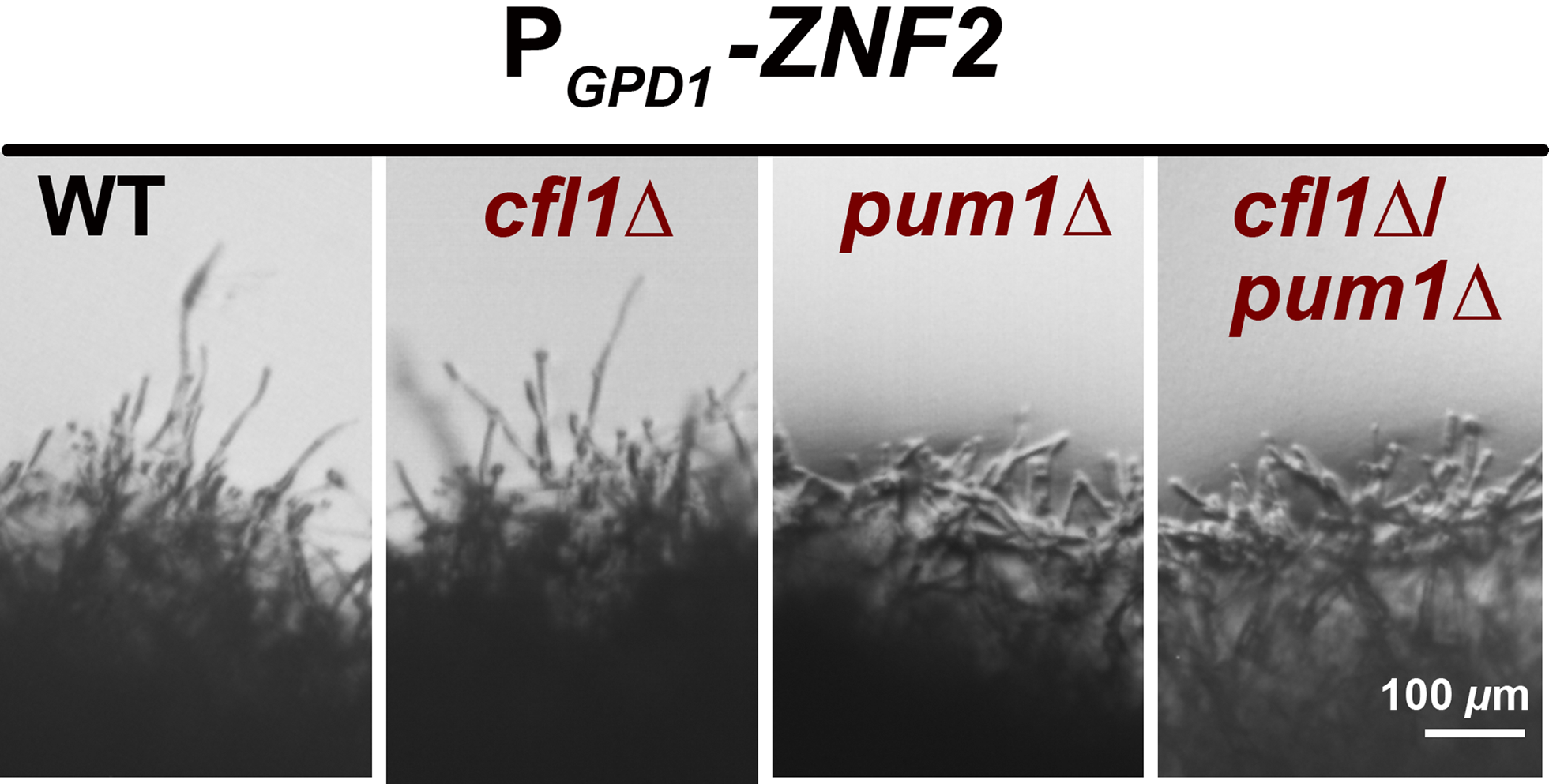

Supplement: Figure S5 — The overexpression of ZNF2 cannot bypass the requirement for PUM1 in promoting sustained hyphal growth. The aerial hyphae were extremely sparse in the pum1Δ/cfl1Δ mutant even in response to ZNF2 overexpression, which prohibited further analysis of aerial hyphal length in this strain. By contrast, invasive hyphae were only modestly affected when ZNF2 was overexpressed in this double deletion mutant. The ZNF2 overexpression did not restore the defect in hyphal extension in the absence of Pum1. (TIF) [file ppat.1004185.s005.tif]

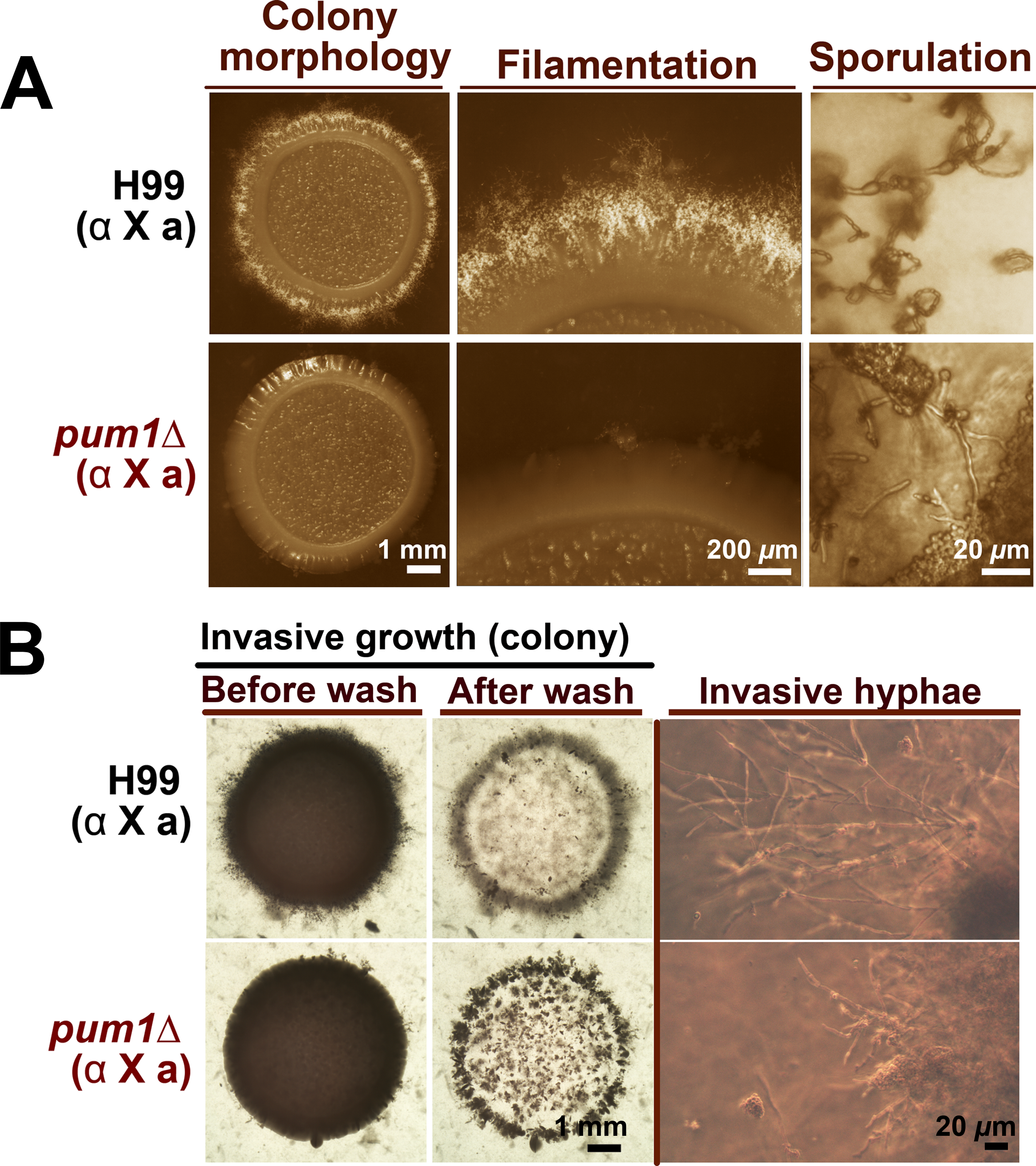

Supplement: Figure S6 — Pum1 orchestrates filamentation and sporulation in the clinical isolate H99 during bisexual mating. (A) Deletion of PUM1 impaired filamentation and abolished sporulation during bisexual mating in the H99 background. (B) Pum1 has a minor role in controlling invasive growth but is required for hyphal extension. (TIF) [file ppat.1004185.s006.tif]
