# Supplementary material for: Morphotype Transition and Sexual Reproduction Are Genetically Associated in a Ubiquitous Environmental Pathogen
Source: PLoS Pathog. 2014 Jun 5;10(6):e1004185. doi: 10.1371/journal.ppat.1004185 (PMC4047104; doi:10.1371/journal.ppat.1004185)
Supplement: Table S3 — Strains used in this study. (DOC) [file ppat.1004185.s009.doc]

**Table S3. Strains used in this study.**

| **Strain name** | **Genotype** | **Source and comments** |
| --- | --- | --- |
| JEC21α | wild type | [1,2] |
| JEC20**a** | wild type | Congenic with JEC21 [1,2] |
| XL877α ^1^ | *ade2* | [3] |
| XL878**a** ^1^ | *lys1* | [4] |
| XT16 **a** ^1^ | *pum1:: NEO^r^, ade2* | This study |
| XT17α ^1^ | *pum1:: NEO^r^, lys1* | This study |
| XL867**a**^1^ | *znf2:: NAT^r^* | [5] |
| XL576α ^1^ | *znf2:: NAT^r^* | [5] |
| XL1417**a** ^1^ | *pum1:: NEO^r^* | This study |
| XL1415α ^1^ | *pum1:: NEO^r^* | This study |
| XT1**a** ^1^ | *Cfl1*::*NEO^r^ pum1:: NEO^r^* | This study |
| XT3α ^1^ | *Cfl1*::*NEO^r^ pum1:: NEO^r^* | This study |
| LW181α ^1^ | P*_CFL1_-CFL1::mCherry:: NEO^r^* | [3] |
| LW188**a** ^1^ | P*_CFL1_-CFL1::mCherry:: NEO^r^* | [3] |
| XL1448 **a** ^1^ | *Cfl1*::*NEO^r^* | [3] |
| XL1449α ^1^ | *Cfl1*::*NEO^r^* | [3] |
| XL353**a**^1^ | *dmc1*::*NAT^r^* | This study |
| XL351α ^1^ | *dmc1*::*NAT^r^* | [6] |
| KN99α ^2^ | wild type | Isogenic with H99 [7] |
| KN99**a** ^2^ | wild type | Congenic with H99/KN99α [7] |
| XX59**a** ^2^ | *pum1:: NEO^r^* | This study |
| XX38α ^2^ | *pum1:: NEO^r^* | This study |
| LW27α ^2^ | P*_CTR4-2_-ZNF2* | [3] |
| XL280α ^3^ | wildtype | [8] |
| XL1359α ^3^ | *cfl1*::*NEO^r^* | [3] |
| XL574α ^3^ | *znf2*::*NAT^r^* | [4] |
| LW593α ^3^ | *fas1:: NEO^r^* | This study |
| XL1346α ^3^ | *fad1:: NEO^r^* | This study |
| RG48α ^3^ | *dha1:: NEO^r^* | This study |
| XL1355α ^3^ | *pum1:: NEO^r^* | This study |
| XT7α ^3^ | *cfl1*::*NEO^r^ pum1:: NEO^r^* | This study |
| XL388α ^3^ | *dmc1*::*NAT^r^* | This study |
| LW1α ^3^ | *znf2*::*NAT^r^* P*_GPD1_-ZNF2:: NEO^r^* | XL574α [4] transformed with pXL1-ZNF2D that bears the *ZNF2* gene with the *GPD1* promoter [9]. |
| LW588α ^3^ | *pum1:: NEO^r^* P*_GPD1_-ZNF2:: NEO^r^* | This study |
| LW591α ^3^ | *cfl1:: NEO^r^* P*_GPD1_-ZNF2:: NEO^r^* | This study |
| LW576α ^3^ | *cfl1*::*NEO^r^ pum1:: NEO^r^* P*_GPD1_-ZNF2:: NEO^r^* | This study |
| LW389α ^3^ | P*_GPD1_-PUM1:: NEO^r^* | This study |
| LW624α ^3^ | P*_CTR4-2_-FAS1::mCherry:: NEO^r^* | This study |
| LW724α ^3^ | P*_CTR4-2_-FAS1(sigP*Δ*)::mCherry:: NEO^r^* | This study |
| XT72α ^3^ | *pum1:: NEO^r^* P*_CFL1_-CFL1::mCherry:: NEO^r^* | This study |
| LW344α ^3^ | *znf2:: NAT^r^* P*_CFL1_-CFL1::mCherry:: NEO^r^* | [10] |
| LW728α ^3^ | P*_FAS1_-FAS1::mCherry:: NEO^r^* | This study |
| XT146α ^3^ | *pum1:: NEO^r^* P*_FAS1_-FAS1::mCherry:: NEO^r^* | This study |
| XT140α ^3^ | *cfl11:: NEO^r^* P*_FAS1_-FAS1::mCherry:: NEO^r^* | This study |
| LW829α ^3^ | P*_DMC1_-DMC1::mCherry:: NEO^r^* | This study |
| XT187α ^3^ | *pum1:: NEO^r^* P*_DMC1_-DMC1::mCherry:: NEO^r^* | This study |
| LW206α ^3^ | P*_GPD1-_CFL1:: NEO^r^* | [3] |
| LW-OE1α ^3^ | P*_GPD1-_*CNAG-00596*:: NEO^r^* | This study |
| LW-OE2α ^3^ | P*_GPD1-_* CNAG-00699*:: NEO^r^* | This study |
| LW-OE3α ^3^ | P*_GPD1-_*CNAG-00849*:: NEO^r^* | This study |
| LW-OE4α ^3^ | P*_GPD1-_*CNAG-00925*:: NEO^r^* | CNAG_00925 encodes Fad1 (This study). |
| LW-OE5α ^3^ | P*_GPD1-_*CNAG-01052*:: NEO^r^* | This study |
| LW-OE6α ^3^ | P*_GPD1-_*CNAG-01121*:: NEO^r^* | This study |
| LW-OE7α ^3^ | P*_GPD1-_*CNAG-01653*:: NEO^r^* | This study |
| LW-OE8α ^3^ | P*_GPD1-_*CNAG-03204*:: NEO^r^* | This study |
| LW-OE9α ^3^ | P*_GPD1-_*CNAG-03650*:: NEO^r^* | This study |
| LW-OE10α ^3^ | P*_GPD1-_*CNAG-03716*:: NEO^r^* | This study |
| LW-OE11α ^3^ | P*_GPD1-_*CNAG-03830*:: NEO^r^* | This study |
| LW-OE12α ^3^ | P*_GPD1-_*CNAG-04373*:: NEO^r^* | This study |
| LW-OE13α ^3^ | P*_GPD1-_*CNAG-04837*:: NEO^r^* | This study |
| LW-OE14α ^3^ | P*_GPD1-_*CNAG-04874*:: NEO^r^* | This study |
| LW-OE15α ^3^ | P*_GPD1-_*CNAG-04944*:: NEO^r^* | This study |
| LW648α ^3^ | P*_GPD1-_* CNAG-05729*:: NEO^r^* | CNAG_05729 encodes Fas1 (This study). |
| LW-OE16α ^3^ | P*_GPD1-_*CNAG-05735*:: NEO^r^* | This study |
| LW-OE17α ^3^ | P*_GPD1-_*CNAG-05778*:: NEO^r^* | This study |
| LW-OE18α ^3^ | P*_GPD1-_*CNAG-06000*:: NEO^r^* | This study |
| LW-OE19α ^3^ | P*_GPD1-_*CNAG-06312*:: NEO^r^* | This study |
| LW-OE20α ^3^ | P*_GPD1-_*CNAG-06346*:: NEO^r^* | This study |
| LW-OE21α ^3^ | P*_GPD1-_*CNAG-06396*:: NEO^r^* | This study |
| LW-OE22α ^3^ | P*_GPD1-_*CNAG-06411*:: NEO^r^* | This study |
| LW-OE23α ^3^ | P*_GPD1-_*CNAG-07203*:: NEO^r^* | This study |
| LW-OE24α ^3^ | P*_GPD1-_*CNAG-07422*:: NEO^r^* | CNAG_07422 encodes Dha1, an antigen protein (3, 6). |

^1^: Strains in JEC21/JEC20 background (serotype D)

^2^: Strains in H99α/KN99**a** background (serotype A)

^3^: Strains in XL280 background (serotype D)

**References**

1. Kwon-Chung KJ, Kozel TR, Edman JC, Polacheck I, Ellis D, et al. (1992) Recent advances in biology and immunology of *Cryptococcus neoformans*. J Med Vet Mycol 30 Suppl 1: 133-142.

2. Heitman J, Allen B, Alspaugh JA, Kwon-Chung KJ (1999) On the origins of congenic *MAT*a and *MAT***a** strains of the pathogenic yeast *Cryptococcus neoformans*. Fungal Genet Biol 28: 1-5.

3. Wang L, Zhai B, Lin X (2012) The link between morphotype transition and virulence in *Cryptococcus neoformans*. PLoS Pathog 8: e1002765.

4. Lin X, Jackson JC, Feretzaki M, Xue C, Heitman J (2010) Transcription factors Mat2 and Znf2 operate cellular circuits orchestrating opposite and same-sex mating in *Cryptococcus neoformans*. PLoS Genet 6: e1000953.

5. Lin X, Jackson JC, Feretzaki M, Xue C, Heitman J (2010) Transcription factors Mat2 and Znf2 operate cellular circuits orchestrating opposite- and same-sex mating in *Cryptococcus neoformans*. PLoS Genet 6: e1000953.

6. Lin X, Hull CM, Heitman J (2005) Sexual reproduction between partners of the same mating type in *Cryptococcus neoformans*. Nature 434: 1017-1021.

7. Nielsen K, Cox GM, Wang P, Toffaletti DL, Perfect JR, et al. (2003) Sexual cycle of *Cryptococcus neoformans var. grubii* and virulence of congenic **a** and a isolates. Infect Immun 71: 4831-4841.

8. Lin X, Huang JC, Mitchell TG, Heitman J (2006) Virulence attributes and hyphal growth of *C. neoformans* are quantitative traits and the *MAT*a allele enhances filamentation. PLoS Genet 2: e187.

9. Hsueh YP, Xue C, Heitman J (2009) A constitutively active GPCR governs morphogenic transitions in *Cryptococcus neoformans*. EMBO J 28: 1220-1233.

10. Wang L, Tian X, Gyawali R, Lin X (2013) Fungal adhesion protein guides community behaviors and autoinduction in a paracrine manner. Proc Natl Acad Sci U S A 110: 11571-11576.
