# Supplementary material for: Morphotype Transition and Sexual Reproduction Are Genetically Associated in a Ubiquitous Environmental Pathogen
Source: PLoS Pathog. 2014 Jun 5;10(6):e1004185. doi: 10.1371/journal.ppat.1004185 (PMC4047104; doi:10.1371/journal.ppat.1004185)
Supplement: Table S4 — Primers used in this study. (DOC) [file ppat.1004185.s010.doc]

**Table S4**. Primers used in this study.

| **Comments** | **Primer name** | **Sequence** |
| --- | --- | --- |
| *PUM1* (JEC21) overexpression | Linlab841 | TGATCTGGCCGGCCGGTTGAAATGGCTACGCAGGGCA |
|  | Linlab842 | GTTTAATTAACCACGAGAGATTAATCACCACCCTT |
| CNAG_00699 overexpression | Linlab1025 | TGATCTGGCCGGCCAGACGATATGCTCGTCAACAAC |
|  | Linlab1026 | GTTTAATTAAAATCGCGTTTATCTATGCTCG |
| CNAG_01052 overexpression | Linlab1027 | TGATCTGGCCGGCCCACCGTTATGTTCCGTCAGACTT |
|  | Linlab1028 | GTTTAATTAAGGATTGCCAGTTAAAGAGTGTTGT |
| CNAG_01653 overexpression | Linlab1039 | TGATCTGGCCGGCCTCTTACTATGATTTTTAATCGTTTC |
|  | Linlab1040 | GTTTAATTAATAAAAACGAATCGTACAGGAGAA |
| CNAG_03204 overexpression | Linlab1045 | TGATCTGGCCGGCCACTCACCATGCTTGCCCACTTCA |
|  | Linlab1046 | GTTTAATTAAGCTGGCCCAAACCATTTTAGAGA |
| CNAG_03405 overexpression | Linlab1049 | TGATCTGGCCGGCCTTACACAATGCCTACCACTCTTG |
|  | Linlab1050 | GTTTAATTAACCTCTCCCTCATGAGCTTTCA |
| CNAG_03650 overexpression | Linlab1051 | TGATCTGGCCGGCCCTCCATAATGAAGATCTTCAGCAGTT |
|  | Linlab1052 | GTTTAATTAAGCTTTCCCGTGGTTTATCTAGCTA |
| CNAG_03716 overexpression | Linlab1053 | TGATCTGGCCGGCCTCTCAATATGCTATTCTCATCTCTT |
|  | Linlab1054 | GTTTAATTAACAGTAACAGAAACACTTCGAAAG |
| CNAG_03857 overexpression | Linlab1057 | TGATCTGGCCGGCCTACCAATATGAAGCTCACTCTC |
|  | Linlab1058 | GTTTAATTAATAAAAGATAACGAAAATGATTCT |
| CNAG_04373 overexpression | Linlab1059 | TGATCTGGCCGGCCAATAGCCATGCTCACATCCATTA |
|  | Linlab1060 | GTTTAATTAAGAGCATGGTAAATGCCTAAAGTT |
| CNAG_05735 overexpression | Linlab1067 | TGATCTGGCCGGCCCATCCTGATGCTCCTCGCCGCTTT |
|  | Linlab1068 | GTTTAATTAAGCAGCGCGTGCATGCCGTCTTAA |
| CNAG_06000 overexpression | Linlab1069 | TGATCTGGCCGGCCACCTACCATGCTTCCCCTCTC |
|  | Linlab1070 | GTTTAATTAAGATAACCAGTTTGGTCCGTCAAA |
| CNAG_06312 overexpression | Linlab1071 | TGATCTGGCCGGCCTACTACAATGCGCGCTTCCATCGT |
|  | Linlab1072 | GTTTAATTAATGCATACAAAACCGAAGGCCACAT |
| CNAG_06346 overexpression | Linlab1073 | TGATCTGGCCGGCCATCCAAGATGTTCACCAAGATC |
|  | Linlab1074 | GTTTAATTAATAGAACAGCAGAACGCTTTAGTT |
| CNAG_06347 overexpression | Linlab1075 | TGATCTGGCCGGCCAACAAAAATGTTCACCAAGGCTAT |
|  | Linlab1076 | GTTTAATTAAGGGGAAAAGACATCGCTAACTA |
| CNAG_06396 overexpression | Linlab1077 | TGATCTGGCCGGCCAGTTAAAATGAAGGTCGTCGCTCTT |
|  | Linlab1078 | GTTTAATTAACAACCAAAAACCAGGCTAGCAAT |
| CNAG_07203 overexpression | Linlab1079 | TGATCTGGCCGGCCTCTCAGAATGGCGCAAATCGTT |
|  | Linlab1080 | GTTTAATTAACCTCGTTTTAGCCAACAAGGGTAGT |
| CNAG_04944 overexpression | Linlab835 | TGATCTGGCCGGCCTCTTGACATGAGGAGTCCCGGTC |
|  | Linlab836 | GTTTAATTAAAAAACTCACCAGGACATTATAATGT |
| CNAG_04874 overexpression | Linlab888 | TGATCTGGCCGGCCCCTATCGATGTTGACCTTACTTGC |
|  | Linlab889 | GTTTAATTAACTAGGCAGGCTAAAGATTATCTCAG |
| *FAS1* (h99)  overexpression | LinLab759 | TGATCTGGCCGGCCCATCAAGATGCGATTCACCTCCAT |
|  | LinLab760 | GTTTAATTAATGATAACGGGGCTTAAAGAATAGA |
| CNAG_06411  overexpression | LinLab755 | TGATCTGGCCGGCCTAAAATAATGTTCCCTTTCAACTCT |
|  | LinLab756 | GTTTAATTAAGTGATTCCTTTAGCAAGTAATAGAA |
| CNAG_06239  Overexpression | LinLab825 | TGATCTGGCCGGCCTCCCATCATGTCTGCGTCAATT |
|  | LinLab826 | GTTTAATTAAATGTATCATTATCCATCTATGGCAA |
| *DHA1* overexpression | Linlab761 | TGATCTGGCCGGCCACCCGCCATGTTCTCGTCCACTA |
|  | Linlab762 | GTTTAATTAACGTCGATATACATCTTACAGCTGGA |
| CNAG_05778  overexpression | Linlab831 | TGATCTGGCCGGCCGCCCACTATGCCCGCTAACTTTA |
|  | Linlab832 | GTTTAATTAATTGGTTAAAATGATACCTAAAATTGTC |
| CNAG_00596  overexpression | Linlab765 | TGATCTGGCCGGCCCAACACGATGATACGCCTCAACATC |
|  | Linlab766 | GTTTAATTAACCATCGGACATAGAAGATTAGATGA |
| CNAG_00925 overexpression | Linlab763 | TGATCTGGCCGGCCTGTCAGGATGCGTTTTACTTCTATC |
|  | Linlab764 | GTTTAATTAATTGCTGGTTCCAGTACTTAAAGGAT |
| CNAG_01121  overexpression | Linlab833 | TGATCTGGCCGGCCCAGCAACATGTTCTTCACATATCTC |
|  | Linlab834 | GTTTAATTAACAAAAAGAGAAGAATTATCTGCTGC |
| *FAS1* (JEC21)  overexpression | Linlab1489 | TGATCTGGCCGGCCCATCAAGATGCGATTCACCTCCAT |
|  | Linlab1490 | GTTTAATTAACACTCTTCAATTTTGATAACGGGACTT |
| *TEF1* QPCR | LinLab329 | CGTCACCACTGAAGTCAAGT |
|  | LinLab330 | AGAAGCAGCCTCCATAGG |
| *MAT2* QPCR | LinLab975 | GCTCCTCGCTACATCTCCTCA |
|  | LinLab976 | TGTTTCGGTCTACGATACCAGTT |
| *STE3α* QPCR | LinLab1668 | TAGCGGAGCGGACTGGAAAGA |
|  | LinLab1669 | CTCGACCGAGACGGCAATCATTA |
| *STE6α* QPCR | Linlab1670 | GCGAATCCACCACCGAATCAATC |
|  | Linlab1671 | CGACGACTGCAACGCACTCT |
| *MF1α* QPCR | LinLab1267 | ATCTTCACCACCTTCACTTCT |
|  | LinLab1268 | CTAGGCGATGACACAAAGG |
| *PUM1 QPCR* | Linlab1343 | TTGTTGGAGGATTTCAGGTTGA |
|  | Linlab1344 | GTCTTCAGGAGTGGCGGTTT |
| *FAS1 QPCR* | Linlab1440 | GTTGCCGCCCTTCCCTTCAT |
|  | Linlab1441 | CCCTGGTTTGGAGTTCGGTGATAA |
| *DMC1 QPCR* | Linlab1666 | AACCAGTTCCTCGCACGTCTTC |
|  | Linlab1667 | TGGGTCAGCCTGTACTTGATTGG |
| *P_FAS1_-FAS1-mCherry-3’utr* | Linlab1283 | TCAGCGGCCGCTGCAGCACCAATGACGAGTTCAT |
|  | Linlab1284 | GCCCTTGCTCACCATAAGAATAGAAAGAAACCCACCAAGGA |
|  | Linlab1285 | TCCTTGGTGGGTTTCTTTCTATTCTTATGGTGAGCAAGGGC |
|  | Linlab864 | GTTTAATTAATTACTTGTACAGCTCGTCCA |
|  | Linlab 1574 | CACTCTTCAATTTTGATAACGGGACGGCCTACTTGTACAGCTC  GTCCATGC |
|  | Linlab 1575 | GCATGGACGAGCTGTACAAGTAGGCCGTCCCGTTATCAAAATT  GAAGAGTG |
|  | Linlab 1576 | GTTTAATTAAGCTGCTGAAAACGGCAAAATAA |
| *P_DMC1_-DMC1-mCherry-3’utr* | Linlab 1680 | TCAGCGGCCGCGAGCTTGAGATACCGCTCTCCTCGAA |
|  | Linlab 1681 | GCCCTTGCTCACCATGCTTGGGTCTTCCCAGCCCCTAA |
|  | Linlab 1682 | TTAGGGGCTGGGAAGACCCAAGCATGGTGAGCAAGGGC |
|  | Linlab864 | GTTTAATTAATTACTTGTACAGCTCGTCCA |
|  | Linlab 1754 | GGAGTCAAAATGAGTAGCAATCCGTCTACTTGTACAGCTCGTCCATGC |
|  | Linlab 1755 | GCATGGACGAGCTGTACAAGTAGACGGATTGCTACTCATTTTGACTCC |
|  | Linlab 1756 | GTTTAATTAAAATGAGCAGGAGGGTCTGTGAAA |
| *P_PUM1_-PUM1-mCherry* | Linlab1088 | TCAGCGGCCGCCACTTCTTACCTTCACTCTTTGCT |
|  | Linlab1092 | GCCCTTGCTCACCATCCACCCTTCAGTTTCGTCCTT |
|  | Linlab1091 | AAGGACGAAACTGAAGGGTGGATGGTGAGCAAGGGC |
|  | Linlab864 | GTTTAATTAATTACTTGTACAGCTCGTCCA |
| P*_CTR4-2_-FAS1(sigP*Δ*)-mCherry* | Linlab 1581 | TGATCTGGCCGGCCCATCAAGATGGCTCCTCTCGTGGAGAGCGCTT |
|  | Linlab1284 | GCCCTTGCTCACCATAAGAATAGAAAGAAACCCACCAAGGA |
|  | Linlab1285 | TCCTTGGTGGGTTTCTTTCTATTCTTATGGTGAGCAAGGGC |
|  | Linlab864 | GTTTAATTAATTACTTGTACAGCTCGTCCA |
| *PUM1* deletion  (JEC21/JEC20/XL280) | Pum1-D-L-F | CCCGACCAGGAGGCGTC |
|  | Pum1-D-L-M13 | CTGGCCGTCGTTTTACCGTCGTTGCCGTCGTAATC |
|  | Pum1-D-R-M13 | GTCATAGCTGTTTCCTGCCCACATCGCCTGAGTTG |
|  | Pum1-D-R-R | GGGGCCTTGATGTCGTACC |
|  | Pum1-D-L-far | CGCATCTCCCTCACATCG |
| *PUM1* deletion  (H99) | XLIN 1 | GCTCGTGGCTGCTACATG |
|  | XLIN 2 | CTGGCCGTCGTTTTACGCTGATGGTGTGAGATGG |
|  | XLIN 3 | GTCATAGCTGTTTCCTGGTGCACGCTCATTTCTTGG |
|  | XLIN 4 | GAGACCCTGGGTTCGG |
|  | XLIN 5 | GGATGAACAAGTCTGTCTCC |
| *FAS1* deletion  (JEC21/JEC20/XL280) | Linlab1315 | CACCAGCGACCAACCACCACAAATG |
|  | Linlab1316 | CTGGCCGTCGTTTTACAAGGGAAGGGCGGCAACGAACAT |
|  | Linlab1317 | GTCATAGCTGTTTCCTGTGAGGAGGAGCGGATAGCGTCTACT |
|  | Linlab1318 | GTCTGACTGTGCTCACTGCGGTAAC |
| *FAD1* deletion  (XL280) | Fad1-D-L-F | CATGGACGCCATTTCGG |
|  | Fad1-D-L-M13 | CTGGCCGTCGTTTTACCCCCGCATCGGTGCTC |
|  | Fad1-D-R-M13 | GTCATAGCTGTTTCCTGGCTTCTCGGCGGTCTCC |
|  | Fad1-D-R-R | CGATCCTTGTCCCGCTAAC |
|  | Fad1-D-L-far | GGGAGCTGCTCTGTGAAGG |
| *DHA1*deletion (XL280) | Linlab1228 | CTGGCCGTCGTTTTACCGTTGTTGTTGTCGTGAAAG |
|  | Linlab1229 | GTCATAGCTGTTTCCTGCGGCAAAAATCTGTATCCC |
|  | Linlab1230 | AGATTGACATTGTGGTCCTCA |
|  | Linlab1231 | GCTCTCGGCTACAATACTGG |
